# Laboratory Colonisation and Genetic Bottlenecks in the Tsetse Fly *Glossina pallidipes*

**DOI:** 10.1371/journal.pntd.0002697

**Published:** 2014-02-13

**Authors:** Marc Ciosi, Daniel K. Masiga, Charles M. R. Turner

**Affiliations:** 1 Molecular Biology and Bioinformatics Unit, ICIPE, Nairobi, Kenya; 2 Institute of Infection, Immunity & Inflammation, University of Glasgow, Glasgow, United Kingdom; National Institute of Allergy and Infectious Diseases, United States of America

## Abstract

**Background:**

The IAEA colony is the only one available for mass rearing of *Glossina pallidipes*, a vector of human and animal African trypanosomiasis in eastern Africa. This colony is the source for Sterile Insect Technique (SIT) programs in East Africa. The source population of this colony is unclear and its genetic diversity has not previously been evaluated and compared to field populations.

**Methodology/Principal Findings:**

We examined the genetic variation within and between the IAEA colony and its potential source populations in north Zimbabwe and the Kenya/Uganda border at 9 microsatellites loci to retrace the demographic history of the IAEA colony. We performed classical population genetics analyses and also combined historical and genetic data in a quantitative analysis using Approximate Bayesian Computation (ABC). There is no evidence of introgression from the north Zimbabwean population into the IAEA colony. Moreover, the ABC analyses revealed that the foundation and establishment of the colony was associated with a genetic bottleneck that has resulted in a loss of 35.7% of alleles and 54% of expected heterozygosity compared to its source population. Also, we show that tsetse control carried out in the 1990's is likely reduced the effective population size of the Kenya/Uganda border population.

**Conclusions/Significance:**

All the analyses indicate that the area of origin of the IAEA colony is the Kenya/Uganda border and that a genetic bottleneck was associated with the foundation and establishment of the colony. Genetic diversity associated with traits that are important for SIT may potentially have been lost during this genetic bottleneck which could lead to a suboptimal competitiveness of the colony males in the field. The genetic diversity of the colony is lower than that of field populations and so, studies using colony flies should be interpreted with caution when drawing general conclusions about *G. pallidipes* biology.

## Author Summary

There is only one mass reared laboratory colony of *Glossina pallidipes*, a vector of human African trypanosomiasis and arguably the main vector of animal African trypanosomiasis in eastern Africa. This colony is the main one used for basic research on this species and is intended to be used for Sterile Insect Technique (SIT) programs for control of field populations. The origins of this colony are not clear and the extent to which it is genetically representative of the species is unknown. Using population genetics analyses to compare the current colony with two potential source populations we have shown that the colony is from the Kenya/Uganda border and that its foundation and establishment in the laboratory were associated with a genetic bottleneck, i.e. reduction of genetic variation due to increased genetic drift in a population of reduced size. As a consequence, the genetic diversity of the colony is lower than that of *G. pallidipes* field populations.

## Introduction

Tsetse flies are vectors of African trypanosomes, parasites that cause human and animal African trypanosomiases. Tsetse control is one of the main methods used to combat the disease [1]. To be efficient, tsetse control attempts should involve a combination of methods that are efficient at both high and low population densities [2]. The sterile insect technique (SIT) is particularly important for control as it is one of the few control methods that is efficient at low densities [2]. SIT is effected by the sequential mass release of sterile males generated from a laboratory colony into an infested area. When the sterile males outnumber the wild males they mate more successfully with wild females that will then produce no offspring. SIT has proven to be an efficient control technique in different parts of the world mainly in pests of crops but was also successfully used to eradicate the New World screwworm *Cochliomyia hominivorax*, a veterinary pest, in North and Central America [reviewed in 3].

To be successful, SIT programmes need to overcome a number of potential genetic difficulties. Firstly, genetic and phenotypic differentiation can cause mating barriers between wild populations which can make SIT less effective depending on the geographical origin of the sterile insects [4]. Second, the establishment of a laboratory colony for SIT is likely to be associated with strong selection pressure toward laboratory adaptation and loss of genetic diversity compared to the field population of origin. Third, as SIT often involves the release of males, the use of an imbalanced sex ratio within the colony (a common practice) is likely to increase genetic drift in the colony and thus loss of genetic diversity compared with the original field population. Laboratory adaptation and genetic diversity loss can be associated with a loss of field competitiveness [4], [5] which can limit the efficiency of SIT.

SIT has proven useful in tsetse control, the most striking example being the eradication of *Glossina austeni* from Unguja Island (Zanzibar), Tanzania through an area-wide integrated tsetse eradication project terminated by a phase of SIT [6]. Following this success, new tsetse SIT programs have started including one targeting *Glossina pallidipes*, a vector of human African trypanosomiasis [7]–[9] and arguably the main vector of animal African trypanosomiasis in eastern Africa [10]. Pilot releases of sterile *G. pallidipes* males are about to be performed [11].

The IAEA *G. pallidipes* colony is one of the very few laboratory colonies of this species worldwide and the only one with which mass rearing has been achieved to date. This is the reason why it has been used to start mass rearing in all the SIT Facilities that are currently rearing *G. pallidipes* [12], [13]. The IAEA *G. pallidipes* colony is generally considered to have been established from the laboratory colony of the University of Amsterdam, the Netherlands which was in turn established from wild pupae collected from Lugala, Uganda in 1975 [14]. Several details associated with the origin of the colony are unclear however. For example, the ‘start date’ for the IAEA colony varies between publications [see 15], [16]–[18], [19 for different dates assumed for the initiation of the IAEA colony]. Moreover, an IAEA report from March 1987 indicate that “A new colony of *G. pallidipes* was initiated from pupae kindly donated by the Tsetse Research Laboratory, Bristol, England.” and this is believed to have been the start of the current IAEA colony [12], [17], [20]. This potential other origin is important because the *G. pallidipes* sent to IAEA in 1987 may have originated from northern Zimbabwe [21]. In summary, published accounts do not allow the identification of a single field population as the source of the current *G. pallidipes* IAEA colony and cannot rule out the possibility of admixture between colonies of Zimbabwean and Ugandan origin. This lack of clarity is potentially important because of the high genetic differentiation between *G. pallidipes* populations from Uganda and Zimbabwe [22]. If the IAEA colony was the result of an admixture between those populations it would harbour much of the genetic diversity present in the species. This could be an advantage for future SIT programs as it could limit potential mating barriers with field populations targeted by SIT control.

Molecular markers and population genetics can be used to reconstruct the demographic history of populations, thereby providing insight into aspects of population establishment and colonisation such as founder effect, population bottleneck and demographic expansion [e.g. 23], [24]–[26]. In that context, the recently developed Approximate Bayesian Computation (ABC) [27]–[29] has proven useful to identify complex and unexpected colonisation histories [30], [31]. ABC allows the quantitative comparison of complex demographic or evolutionary scenarios and the estimation of parameters of interest based on molecular and historical data. It has been shown to be a powerful method to compare complex demographic and phylogeographic scenarios based on population genetics data sets [e.g. 23], [30], [32]–[34].

We examined here the genetic variation within and between the IAEA colony and its potential source populations in north Zimbabwe and the Kenya/Uganda border to retrace the demographic history of the IAEA colony. We performed classical population genetics analyses and also combined historical information and microsatellite data in a quantitative analysis of the genetic variation using ABC methods. We addressed the following specific questions: (i) Does the IAEA colony result from an admixture between the two potential source populations or originate from a single source? (ii) What is the level of genetic drift associated with laboratory colonisation and the rearing of the colony between its foundation and the present?

## Materials and Methods

### Sample collection and DNA extraction

*G. pallidipes* were obtained from the IAEA Seibersdorf colony and from its potential source populations (Rukomeshi, Zimbabwe and the Uganda/Kenya border, Table 1). Samples of the IAEA colony obtained in 2012 (30 flies) and 2013 (26 flies) were used to better represent the contemporary demographic stochasticity within the colony. The sample (31 flies) used to represent the Uganda/Kenya border population was collected in 2000 in Kapesur near Busia, Kenya. This location is adequate as there is no genetic differentiation between sites within this area [35]. The Rukomeshi sample (34 flies), collected in 2006, was previously analysed for the presence and genetic diversity of the salivary gland hypertrophy virus by Kariithi et al [36]. Flies sampled in Busia were dried after collection and stored in 95% ethanol. IAEA colony specimens were stored in 95% EtOH. For Busia and IAEA colony samples, DNA extractions were carried out from the abdomen of each fly using the Qiagen DNeasy blood and tissue kit following the manufacturer indications and using a final elution of 100 µl. DNA extractions from Rukomeshi sample were carried out as indicated in Kariithi et al [36].

**Table 1:** *G. pallidipes* samples used in this study plus HWE tests.

Area/country	Sites/colony	Collection date	Latitude(°N), Longitude(°E)	Sample size	HWE	*F*_IS_
**North Zimbabwe**	Rukomeshi	2006	−16.13, 29.40	34	<0.01	0.126
**Uganda/Kenya border**	Busia	April 2000	0.61, 34.30	31	0.906	−0.025
**Seibersdorf, Austria**	IAEA	March 2012	-	30	0.997	−0.090
		April 2013	-	26	0.999	0.012

Sample size: number of individuals genotyped per sample. HWE: p-value of the test for deviation from Hardy–Weinberg equilibrium.

Distinguishing between *G. pallidipes* and *G. fuscipes* can prove challenging when specimens have been stored in 95% EtOH because of the alteration of colours that are the main characteristics that allow distinguish between these two species [37]. To make sure no confusion was made at sampling between *G. pallidipes* and *G. fuscipes fuscipes* in Busia (where these species are sympatric), we tested whether the microsatellite locus GmmF10 could be amplified in flies collected in Busia as this marker can be amplified in *G. pallidipes* but not in *G. fuscipes* [38], [39].

### Microsatellite genotyping

15 polymorphic microsatellites loci previously described in the literature [38]–[42] were evaluated for their use in population genetics analyses of *G. pallidipes* using multiplex PCR as described in Supplementary file S1. Loci were combined into multiplex reactions with the help of Multiplex Manager v1.2 [43] in an analysis of 2 millions iterations, a primer complementarity threshold of 7 and a minimum distance between loci of the same dye color of 26 bp. The multiplex reactions were fine tuned by hand. After a validation step (fully described in Supplementary file S1) we ended up using 9 microsatellites loci in two multiplex PCR. Multiplex reaction α contained loci GmmK06, GmmC17, GpC10b, GpC101, GpB115, GpCAG133. Multiplex reaction β contained loci: GmmA06, GpA19a and GpC26 (Table S1). This resulted in a primer complementary threshold of 6 within multiplex reactions and of a minimum distance between loci of the same dye colour of 58 bp. Multiplex PCR were carried out in a total volume of 10 µl containing 2 µl of template DNA solution, 1X Qiagen Multiplex PCR mix and 0.2 µM of each primers except for locus GpC10b (0.3 µM of each primers). Forward PCR primers 5′ labelled with a fluorescent dye were used to allow the PCR products to be detected on an automated DNA sequencer. The PCR cycling conditions for both multiplex PCRs were (95°C, 15 min); 25 cycles of (94°C, 30 s), (55°C, 90 s) and (72°C, 60 s); (60°C, 30 min). 1 µl of a 1/20 or 1/30 dilution of the multiplex PCR products were analysed by electrophoresis in combination with the GeneScan-500 LIZ size standard (Applied Biosystems) by DNA Sequencing & Services (MRCPPU, College of Life Sciences, University of Dundee, Scotland, www.dnaseq.co.uk) using Applied Biosystems Big-Dye Ver 3.1 chemistry on an Applied Biosystems model 3730 automated capillary DNA sequencer. The size estimation of amplified microsatellites was performed using GeneMarker v2.2.0 (SoftGenetics). The Excel Macro Autobin v0.9 [44] was then used on the raw data set of amplified microsatellites sizes to automatically detect relevant gaps in size and help delimit allele “bins” (Table S2). The allele “bins” defined using Autobin were then used within GeneMarker to automatically bin the alleles. Each peak was then checked manually.

Microsatellite data are available from the Dryad Digital Repository: http://dx.doi.org/10.5061/dryad.bt612

### Genetic variation within samples

Genetic variation within samples was assessed using the mean number of alleles per locus (*Na*) and the mean expected heterozygosity (*H*) [45] computed with Geneclass 2 ver. 2.0.h [46]. The coefficient of inbreeding *F*_IS_ was estimated with Genepop on the Web [47], [48]. For comparisons of *Na* values between samples, allelic richness (*AR*) was estimated on the basis of minimum sample size with Fstat 2.9.3.2 [49]. The significance of differences in *AR* and *H* between samples was assessed with the nonparametric Friedman and Wilcoxon sign rank tests (with the locus as a repetition unit). Deviation from Hardy–Weinberg equilibrium (HWE) was assessed with the probability test approach, using Genepop on the Web.

### Genetic variation between samples

Exact tests for pairwise genic differentiation [50] were performed with Genepop on the Web [47], [48]. The significance levels of those tests were corrected with Benjamini and Hochberg's [51] false discovery rate procedure when necessary as those tests can involve non orthogonal and multiple comparisons. Genetic differentiation between pairs of samples was summarised by Weir and Cockerham's [52] estimator of pairwise *F*_ST_ using Genepop on the Web.

### Bottleneck tests

For each field population and laboratory colony, tests for a recent reduction in population size in the last 2*Ne*−4*Ne* generations were performed using the program Bottleneck 1.2 [53], [54]. These analyses were carried out assuming a generalised stepwise mutation (GSM) with a variance of 0.36 [31], [55], [56]. One-tailed Wilcoxon sign-rank tests were used to determine whether observed heterozygosity deviates from expectations at mutation-drift equilibrium. Estimations were based on 10000 replications. Reductions in population size were also tested using the “modeshift” indicator of the distortion of allele frequency classes' distributions [57].

### Assignment of the IAEA individuals to the potential source populations

Two approaches were used. In the first, we calculated the mean multilocus individual assignment likelihood of each IAEA sample *i*, to each sample of possible source populations *s* [hereafter denoted *L* _i→s_], [ see 58,59] with Geneclass 2 ver. 2.0.g [46]. For each IAEA sample, the most probable source population was then identified as that with both the highest *L*_i→s_ value and the lowest *F*_ST_ value with the source population considered [24], [60].

The second method allowed the concomitant assignment of individuals and inference of potential admixture. This clustering approach, implemented in Structure 2.3.4 [61] was used to evaluate the contribution of the Rukomeshi and Busia populations to the current IAEA colony. Individual multilocus genotypes were used to infer clusters of individuals within which deviation from HWE and linkage disequilibria are minimized. The microsatellite data were converted from Genepop to Structure format using the software Create v.1.37 [62]. Ten replicate runs for each prior value of the number (*K*) of clusters, set between 1 and 5, with a burn-in of 2×10^5^ iterations followed by 10^6^ iterations. The admixture model of ancestry together with the correlated allele frequencies model were used [63] and no account was taken *a priori* on the origin (Busia, Rukomeshi or IAEA) of each individuals, i.e. individuals were clustered only on the basis of their multilocus genotypes. Default values were maintained for all other parameters. *K* was estimated as the value leading to the highest likelihood for the data P(X|*K*) and with the Δ*K* statistics of Evanno et al. [64] with Structure Harvester Web v0.6.93 [65].

### Inferring the past demography of the laboratory colony using Approximate Bayesian Computation

We applied an Approximate Bayesian Computation (ABC) approach to infer the demographic history of the *G. pallidipes* IAEA colony and field populations under study. Microsatellite data were combined with prior information on the history and demography of those populations. Analyses were performed with Diyabc v 1.0.4.46 [66], [67]. Briefly, in an ABC analysis, summary statistics of each simulated dataset are recorded, together with the label of the scenario used for the simulation. Euclidian distances between each simulated dataset and the observed dataset are computed. These distances are then used to estimate the posterior probabilities of the scenarios and posterior probability distributions of the parameters. In each of the three analyses described below and in Supplementary file S2, 10^6^ datasets were simulated for each competing scenario using parameter values drawn from prior distributions and assuming equiprobability of each scenario *a priori*. The simulated datasets had the same characteristics (number of samples, individuals, loci, characteristics of the microsatellite loci) as the observed dataset.

Genetic variation was summarised using a set of summary statistics traditionally used in ABC for each population and each population pair [23], [34], [67]: mean number of alleles, mean gene diversity, mean allele size variance and mean M index across loci [68], pairwise *F*_ST_ [52], mean individual assignment log-likelihoods of individuals from population *i* assigned to population *j* (*L*_i→j_) and the maximum likelihood estimates for admixture proportions [69]. In analyses 1 and 2, four summary statistics were used while there were 54 in analysis 3 (Table S3).

In analysis 1 (Supplementary file S2) we focused on the Busia population in order to correctly model the demographic history of this population when analysing the IAEA colony history. This is of importance as the Busia population may have experienced a genetic bottleneck due to tsetse control [70] or to the destruction of the tsetse habitat associated with the increase of the human population between the foundation of the IAEA colony (1975) and the sampling of the Busia population. If such a bottleneck occurred it is important to take it into account when performing inferences on the demographic history of the IAEA population.

In analysis 2 (Supplementary file S2) we focused on the demography of the Rukomeshi population between the establishment of the IAEA colony and the sampling of the Rukomeshi flies in 2006. Unlike for the Busia population, there is no record of any tsetse control program in Rukomeshi area between 1975 and 2006. However, a field trial of a tsetse control technique has been carried out in Rukomeshi in 1991 and could have decreased the size of the *G. pallidipes* population temporarily [71].

**Figure 1:**
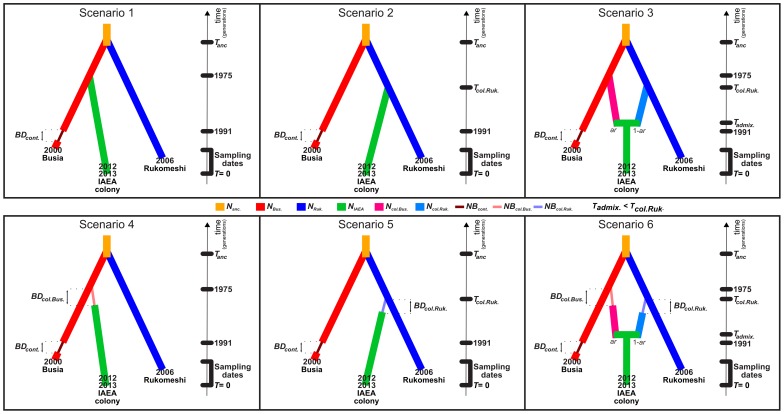
Competing scenarios considered in the ABC analysis of IAEA colony past demography (analysis 3). The demography of the Busia and Rukomeshi populations was determined as described in Supplementary file S2. In all scenarios, the two potential source populations merged *T_anc._* ago into an unsampled ancestral population and a bottleneck started in Busia in 1991 and lasted *BD*_cont._ generations. Scenario 4, 5 and 6 are respectively variations of scenarios 1, 2 and 3 in which a genetic bottleneck (of duration *BD*_col.Bus._ or *BD*_col.Ruk._) is associated with the laboratory colonisation. In scenarios 1, 2, 4 and 5 the IAEA colony has a single population of origin. In scenarios 1 and 4, the IAEA colony was founded from the Busia population in 1975 while in scenarios 2 and 5, it was founded from the Rukomeshi population *T_col.Ruk._* generations ago. In scenario 3 and 6 the IAEA colony originates from an admixture between unsampled colonies of Busia and Rukomeshi origin that were respectively founded from the Busia population in 1975 and from the Rukomeshi population *T_col.Ruk._* generations ago. When admixture occurs, the admixture rate *ar* is the proportion from unsampled Busia colony that contributed to the admixed IAEA colony.

The IAEA colony demography and origin were examined in analysis 3, taking into account the scenarios selected in analysis 1 and 2. The IAEA colony was considered to originate from a single source, Busia or Rukomeshi, or from an admixture between both. Each of those three scenarios were considered with or without the possibility of a bottleneck associated with the laboratory establishment of the IAEA colony, giving a total of 6 competing scenarios (Figure 1). The analyses were performed using parameter values drawn from the prior distributions described in Table 2.

**Table 2:** Prior distributions of the parameters used in the ABC analyses.

Parameters	Prior distributions
*N*_anc._, *N*_Bus._, *N*_Ruk._	Uniform [500; 20000]
*N*_IAEA_, *N*_col.Bus._, *N*_col.Ruk._	Uniform [1; 20000]
*NB_col._*_Bus._, *NB_col._*_Ruk._, *NB*_cont._	Uniform [1; 100]
*T_anc._* (generations before present)	Uniform [500; 25000]
*T_col.Ruk._*	Uniform [1980; 1983]
*T_admix._*	Uniform [1981; 1990]
*BD*_cont._	Uniform [1], [47]
*BD*_col.Bus._	Uniform [1], [15]
*BD*_col.Ruk._	Uniform [1], [25]
*ar*	Uniform [0.1; 0.9]
Mutational model	GSM+SNI with default parameters
Number of generations per year	5*

**Note:** *N*_anc._: effective population size (*Ne*) of the ancestral population. *N*_Bus._: *Ne* of the Busia population. *N*_Ruk._: *Ne* of the Rukomeshi population. *N*_IAEA_: *Ne* of the IAEA colony. *N*_col.Bus._: *Ne* of the unsampled colony of Busia origin. *N*_col.Ruk._: *Ne* of the unsampled colony of Rukomeshi origin. *NB_col._*_Bus._: *Ne* of the colony of Busia origin (IAEA in scenario 4 and unsampled in scenario 6) during the bottleneck associated with its establishment that started in 1975 lasted *BD*_col.Bus._ generations. *NB_col._*_Ruk._: *Ne* of the colony of Rukomeshi origin (IAEA in scenario 5 and unsampled in scenario 6) during the bottleneck associated with its establishment that started at *T_col.Ruk._* and lasted *BD*_col.Ruk._ generations. *NB*_cont._: *Ne* of the Busia population during the bottleneck associated with the tsetse control that started in 1991 and lasted *BD*_cont._ generations. *T_anc._*: number of generations between present and the merge of the two potential source populations an unsampled ancestral population. *T_admix._*: date of the admixture. *ar*: admixture rate. GSM: Generalized Stepwise Mutation model. SNI: Single nucleotide indel mutations.

* : see Supplementary file S2 (Table S7 in Supplementary file S2) for a rationale.

For all the ABC analyses performed, posterior probabilities of the competing scenarios were estimated by polychotomous logistic regression [67] on the 1% simulated datasets closest to the observed dataset. The selected scenario was that obtaining the highest posterior probability with a 95% confidence interval non-overlapping with the second highest probability [30], [31]. The posterior distributions of the demographic parameters were estimated under the selected scenario using a local linear regression on the 1% simulated datasets producing the smallest Euclidian distances to the observed dataset [27], [67]. The median of a posterior distribution was considered as point estimate for a parameter [as in 66], [67].

ABC analyses were performed on simulated pseudo-observed datasets (PODs) to evaluate the ability of our ABC analysis 3 to select the true scenario. For each of the 6 scenarios of the ABC analysis 3 (Figure 1), 100 PODs were simulated using parameter values drawn from the probability distributions identical to the prior distributions (Table 2). Each PODs has the same characteristics (number of samples, individuals, loci) as the observed dataset. For the selection of the scenario, procedures previously described (summary statistics, Euclidian distances, posterior probability estimation) were applied to each POD. Because the scenario used to generate each POD is known, applying the ABC analysis 3 on the PODs allows the estimation of type I and II errors for these analyses. Type I error corresponds to the proportion of PODs for which a scenario is excluded by the ABC analysis while it is actually the true scenario (the one used to generate the PODs). Type II error corresponds to the proportion of PODs for which a scenario is selected while it is not the true one. Low type II error indicates that the results are reliable even when the type I error is large [23].

Using the “model checking” option in Diyabc we evaluated the ability of the selected scenario and of its parameters posterior distributions to generate simulated data that are similar to the observed data set [i.e. evaluation of the “goodness of fit” of the combination of the selected scenario and its parameter posterior distributions, 66]. The procedure was carried out by simulating 10^4^ PODs using the scenario selected in the ABC analysis 3 and parameters values drawn from the posterior distributions of the parameters. Summary statistics distributions corresponding to those 10^4^ PODs were then compared to the observed summary statistics. To reduce the bias introduced by the use of the same set of summary statistics for the ABC analysis and the model checking [66] we added the following summary statistics to the previously used 54 summary statistics: the shared allele distance [72] and the (*δμ*)^2^ distance [(*δμ*)^2^ = (*μ* _A_ – *μ* _B_)^2^, where *μ* _A_ and *μ* _B_ are the “means of allele size in populations A and B”, 73]. That way, 66 summary statistics were used in the “model checking”. The combination of the selected scenario and its parameter posterior distributions would be considered inadequate if many observed summary statistics were not included in the distribution of the summary statistics corresponding to the 10^4^ PODs [66].

### Simulation of the genetic diversity of the source population of the IAEA colony in 1975

In order to evaluate the genetic diversity loss that occurred between the 1975 source population and the current IAEA colony we used Diyabc to simulate the source population in 1975 under the scenario selected by the ABC analysis 3. To take into account the inter-simulation variation, we simulated 100 datasets under the scenario selected by the ABC analysis 3. In addition to the four samples (Busia, Rukomeshi and IAEA 2012 and 2013) included in the ABC analysis 3, a fifth sample corresponding to the 1975 source population was simulated. This sample of the 1975 source population correspond to 26 multilocus genotypes at 9 microsatellite loci having the same characteristics as the ones used to produced the observed data. We simulated 26 multilocus genotypes to be able to directly compare the genetic diversity of the 1975 source population simulated and of the IAEA 2013 sample which is made up of 26 individuals (Table 1). The simulations were performed using the parameter values estimated previously under the scenario selected by the ABC analysis 3 (i.e. the median of the posterior distributions of the parameters). Because the simulations slightly over estimated the genetic diversity of the Rukomeshi sample we downscaled the genetic diversity simulated for Busia 1975 sample accordingly to be conservative in our comparison of IAEA 2013 and the simulated Busia 1975 sample.

## Results

The microsatellite locus GmmF10 could be amplified in each of the flies samples in Busia (see Figure S1) indicating that they are all *G. pallidipes* [38], [39].

### Genetic variation within samples

The complete dataset analysed (121 individuals from Busia, Rukomeshi and the IAEA colony) showed moderate polymorphism with an average of 6.556 (SD = 3.046) alleles per locus over all samples. The number of alleles ranged from 3 at locus GmmC17 to 11 at loci GpC101 and GmmA06. Fifty seven (96.6%) of the 59 alleles observed over all samples were present in the Rukomeshi sample. In Rukomeshi all loci were polymorphic whereas locus GpCAG133 was monomorphic in Busia and both IAEA samples and locus GmmC17 was monomorphic in IAEA 2013. Only the Rukomeshi sample was found to deviate from Hardy-Weinberg equilibrium (Table 1) which was mainly due to a single locus. Only allele frequencies at locus Gpc26b were found to significantly deviate from HWE in Rukomeshi after correction for multiple comparisons (p<0.001). This was associated with heterozygote deficiency (positive *F*_IS_) and could be the results of the presence of null alleles at this locus in Rukomeshi. Most of the analyses including the Structure analysis and the ABC analysis 3 were repeated after excluding locus Gpc26b from the dataset. The results obtained were very similar to the ones obtained with 9 loci (data not shown) and the deviation from HWE at locus Gpc26b was thus considered inconsequential for our inferences. No linkage disequilibrium was detected between loci in any sample.

**Figure 2:**
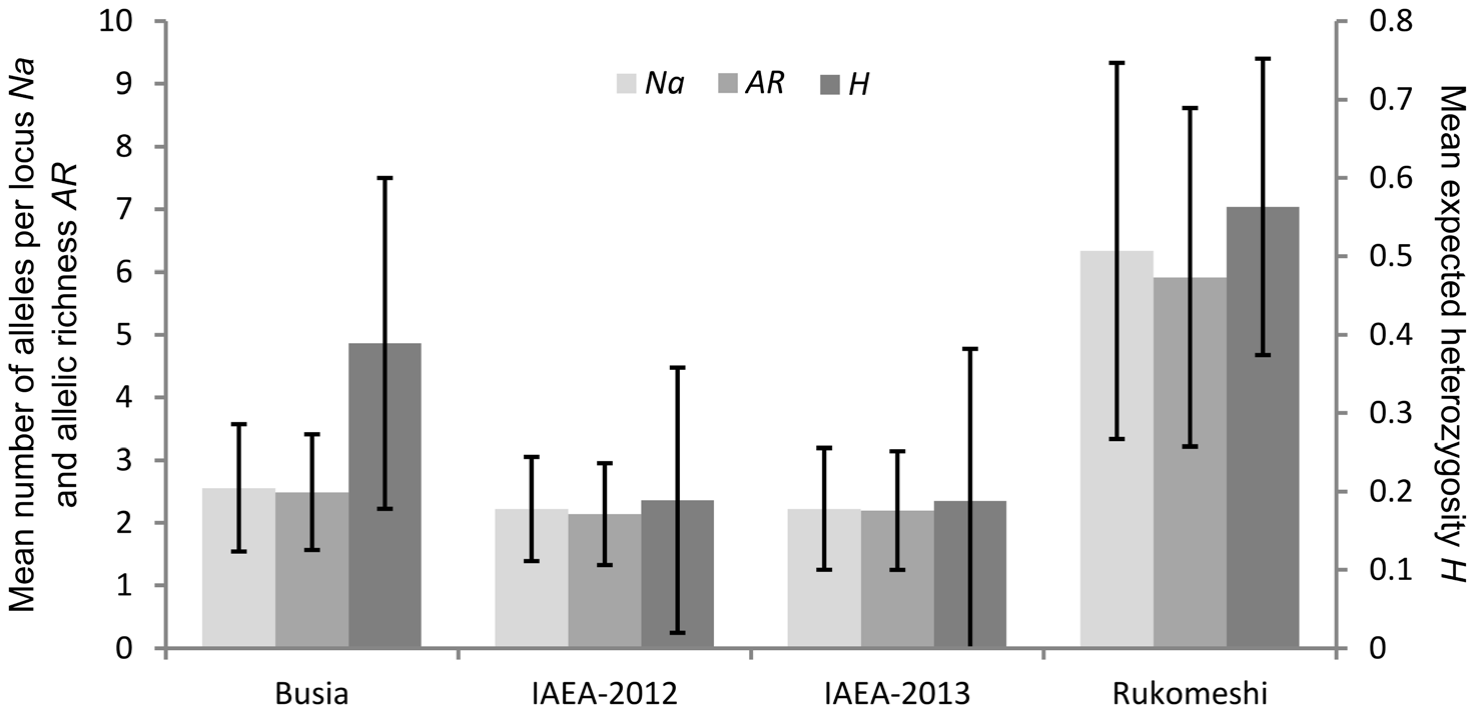
Genetic variations within samples. Error bars indicate the standard deviations across loci. *Na*: average number of alleles per locus. *AR*: allelic richness. *AR* is based on minimum sample size (N = 23 in Friuli for locus DVV-ET1). *H:* mean expected heterozygosity.

**Figure 3:**
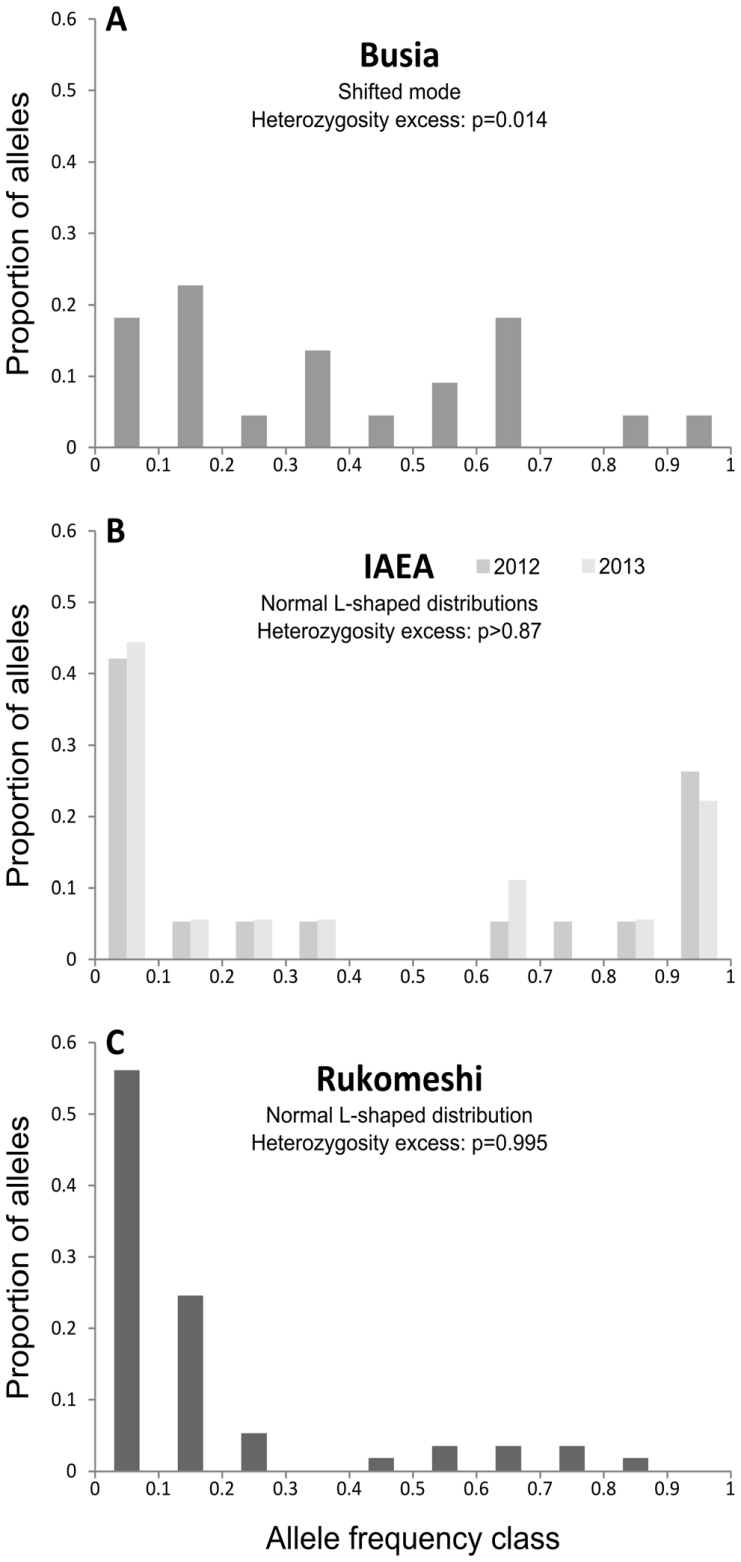
Distribution of the allele frequency classes. (A) in Busia. (B) in the IAEA colony samples from 2012 and 2013. (C) in Rukomeshi. The result of the bottleneck tests (Wilcoxon' tests on heterozygosity excess, and the mode shift tests) are indicated for each panel which correspond to a field population or to the IAEA colony. Heterozygosity excess and/or a shifted mode in the distribution of allele frequency classes indicate a recent reduction in population size.

The mean number of alleles per locus (*Na*) was heterogeneous between samples, ranging from 2.2 [Allelic richness (*AR*) = 2.1] in the IAEA colony to 6.3 (*AR* = 5.9) in Rukomeshi (Figure 2). Similarly, mean expected heterozygosity (*H*) ranged from 0.19 in the IAEA colony to 0.56 in Rukomeshi (Figure 2). *Na* and *AR* were significantly higher in Rukomeshi than the other three samples (*p*<0.01, 9 signed ranks, |W| = 45) while they were no different in Busia and the IAEA samples (*p*>0.05, ≤8 signed ranks, |W|≤18). A different pattern was observed with *H* which was significantly higher in the field populations than in the IAEA colony (*p*≤0.01, ≥8 signed ranks, |W|≥34) but was not different between the two field populations (*p*≥0.05, 9 signed ranks, |W| = 29). The tests for mode shift of the distribution of allele frequency classes and for heterozygosity excess (Figure 3) indicated that the Busia population recently experienced a genetic bottleneck while those tests revealed no evidence of such bottlenecks in Rukomeshi or IAEA populations.

### Genetic variation between samples

Pairwise comparisons between samples showed highly significant genetic differentiation tests and large to very large *F*_ST_ estimates (mean = 0.27, SD = 0.13; Table 3) except for the comparisons between the IAEA colony samples (no genetic differentiation and *F*_ST_ = 0; Table 3). The genetic differentiation between Rukomeshi and the IAEA samples was significantly greater than between the Busia and the IAEA samples (Wilcoxon' sign rank test on pairwise *F*_ST_ per locus, 8 sign ranks, |W|>28, *p*<0.05).

**Table 3:** Genetic differentiation between samples and assignment of the IAEA colony samples into the potential source populations.

	Busia	IAEA-2012	IAEA-2013	Rukomeshi
**Busia**	-	4.0×10^−5^	2.8×10^−5^	-
**IAEA-2012**	**0.133**	-	-	1.9v10^−12^
**IAEA-2013**	**0.130**	0	-	4.3×10^−12^
**Rukomeshi**	**0.276**	**0.406**	**0.389**	-

Pairwise *F*_ST_ are indicated below the diagonal. Significant pairwise genotypic differentiation exact tests are indicated in bold typeface. The mean individual assignment likelihoods of IAEA samples into the potential sources of the colony (*L*_i→s_) are indicated above the diagonal.

We also investigated the mean multilocus individual assignment likelihood of each IAEA sample *i* to each sample of the potential source population *s* (*L*_i→s_) and the results are indicated in Table 3. The highest *L*_i→s_ values for Busia also indicate that this is a more likely source population of the IAEA colony than Rukomeshi.

**Figure 4:**
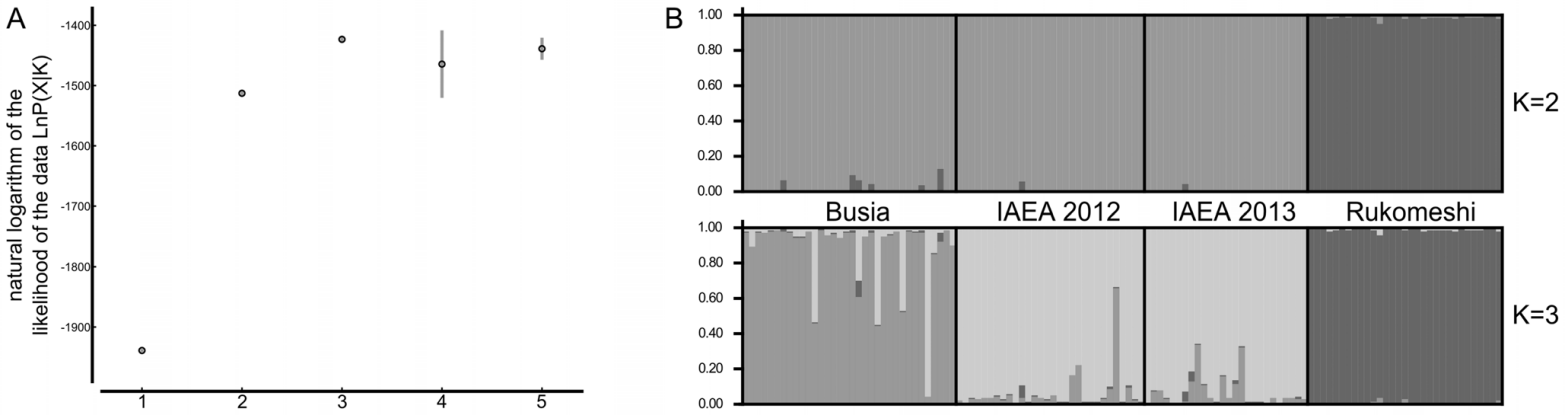
Estimated number of clusters and population structure from the Structure analysis. (A) Mean (±SD) natural logarithm of the likelihood of the data [LnP(X|K)] over 10 Structure replicated runs for each value of the putative number of clusters (K). (B) Estimated population structure from the Structure analysis for K = 2 and 3. Each individual is represented by a vertical line divided into K coloured segments that represent the individual's estimated membership fractions in K clusters. Black lines separate individuals from different samples. Each plot, is based on the highest-probability run (among ten) at K = 2 and 3.

In a third approach, Structure inferred the most likely number of genetic clusters (*K*) to be 2 or 3. The statistic Δ*K* [64] indicated *K* = 2 while the highest likelihood of the data was obtained at *K* = 3 (Figure 4A). At *K* = 2 (Figure 4B, top) all the IAEA individuals belong to the same cluster as the individuals from Busia. Also a very small fraction of the multilocus genotypes of some IAEA individuals clustered with Rukomeshi but this fraction was smaller for the IAEA colony than for Busia and was thus considered uninformative. Similarly, there was no evidence of introgression from Rukomeshi to the IAEA colony at *K* = 3 (Figure 4B, bottom). At *K* = 3 each population (Busia, IAEA colony and Rukomeshi) formed a distinct cluster except for a few Busia individuals that clustered within the IAEA cluster or were admixed between the Busia and the IAEA cluster and vice and versa.

Manual inspection of the allele frequency distributions did however support the possibility of introgression from Rukomeshi into the IAEA colony. All the alleles present in the IAEA samples should normally be present in the sample of its source population and all the alleles of the IAEA colony were found in Rukomeshi but not in Busia. 4 alleles observed in the IAEA colony were absent from the Busia sample and present in Rukomeshi. Two of these alleles (allele 129 at locus GmmK06 and allele 187 at locus GmmC17) were rare in the IAEA colony (allele frequencies ≤0.02) while the other two (alleles 157 and 159 at locus GpA19a) had relatively high frequencies (0.04 to 0.29). However, the interpretation of these data must also take into account the total number of alleles in both Busia and Rukomeshi (23 and 57 respectively). Fisher's exact test shows that the number of private alleles from Busia observed into the IAEA colony (0/23) is not statistically different from the number of private alleles from Rukomeshi (4/57; p = 0.32).

### Inferring the past demography of the laboratory colony using Approximate Bayesian Computation

In the ABC analysis 1 focused on Busia, the scenario with a strong bottleneck (scenario A) obtained a posterior probability of 0.997 (Table 4). The confidence in this scenario choice is good because type II error was small (0.065, Table 4). In the ABC analysis 2 focused on Rukomeshi, the highest posterior probability (0.737) was associated with the scenario with a constant population size (scenario C). However the power of the ABC analysis 2 was low with high type I (0.66) and type II errors (0.275). It should be noted that 84.8% of type I error correspond to the selection of scenario B when scenario C is true and 87.3% of type II error is associated with the selection of scenario C while scenario B is true. To conclude, the results of the ABC analyses 1 and 2 show that a genetic bottleneck occurred recently in Busia but provide no support for such a bottleneck in Rukomeshi.

**Table 4:** Posterior probability of the selected scenarios and confidence in scenario choice in the ABC analyses.

ABC analysis	Number of competing scenarios	Selected scenario	Posterior probability [95% CI]	Type I error	Type II error Mean (min-max)
1 - Busia	3	Scenario 1 (strong bottleneck)	0.997 [0.997,0.998]	0.17	0.065 (0.05–0.08)
2 - Rukomeshi	3	Scenario 3 (constant population size)	0.737 [0.724,0.750]	0.66	0.275 (0.07–0.48)
3 – IAEA colony	6	Scenario 4 (Busia origin with bottleneck)	0.792 [0.756,0.827]	0.25	0.044 (0.05–0.17)

Those results were taken into account and the demography of Busia and Rukomeshi were modelled accordingly in the ABC analysis 3 (Figure 1). To identify the most likely source population of the IAEA colony and estimate the intensity of genetic drift associated with its foundation and establishment, six possible scenarios were considered (Figure 1). Scenario 4 involving a single Busia origin of the IAEA colony and a genetic bottleneck associated with its foundation and establishment was identified as having the highest posterior probability (0.792, Table 4) with small type II error (0.044, Table 4). Although the type I error associated with this analysis was not small (0.25, Table 4), it should be noted that 76% of this corresponds to the selection of scenario 1 when scenario 4 is true (i.e. selection of a scenario in which the source population of the IAEA colony is Busia). The model checking analysis revealed that the combination between scenario 4 and the estimated posterior distributions of the parameters produced simulated data very similar to the observed data. Indeed, only one observed summary statistic [the (*δμ*)^2^ distance, 73] between the IAEA samples (which was not used in our ABC analyses) was in the 5% tail of the distribution of the corresponding simulated statistics. These results give good confidence in the estimation of the parameters associated with scenario 4.

**Figure 5:**
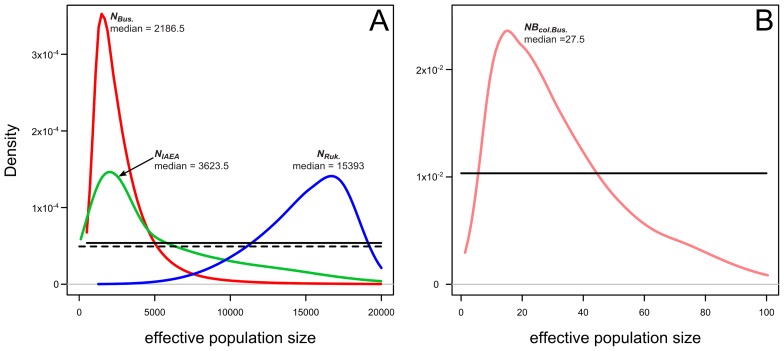
Effective population sizes (*Ne*) estimated from the ABC analysis with scenario 4 (Figure 1). The prior distributions are shown as black lines while the posterior distributions are shown as coloured lines using the same colour code as in Figure 1. The medians of a posterior distribution, considered as point estimate of the parameters, are indicated. Each distribution was obtained from 10000 values. (A) *N*_Bus._: *Ne* of the Busia population. *N*_Ruk._: *Ne* of the Rukomeshi population. *N*_IAEA._: *Ne* of the IAEA colony. The prior distributions for *N*_Bus._ and *N*_Ruk._ are shown as a plain line while the prior distribution for *N*_IAEA._ is shown as a dashed line. (B) *NB_col._*_Bus._: *Ne* of the colony of Busia origin (which is at the origin of the current IAEA colony) during the bottleneck associated with its establishment.

**Figure 6:**
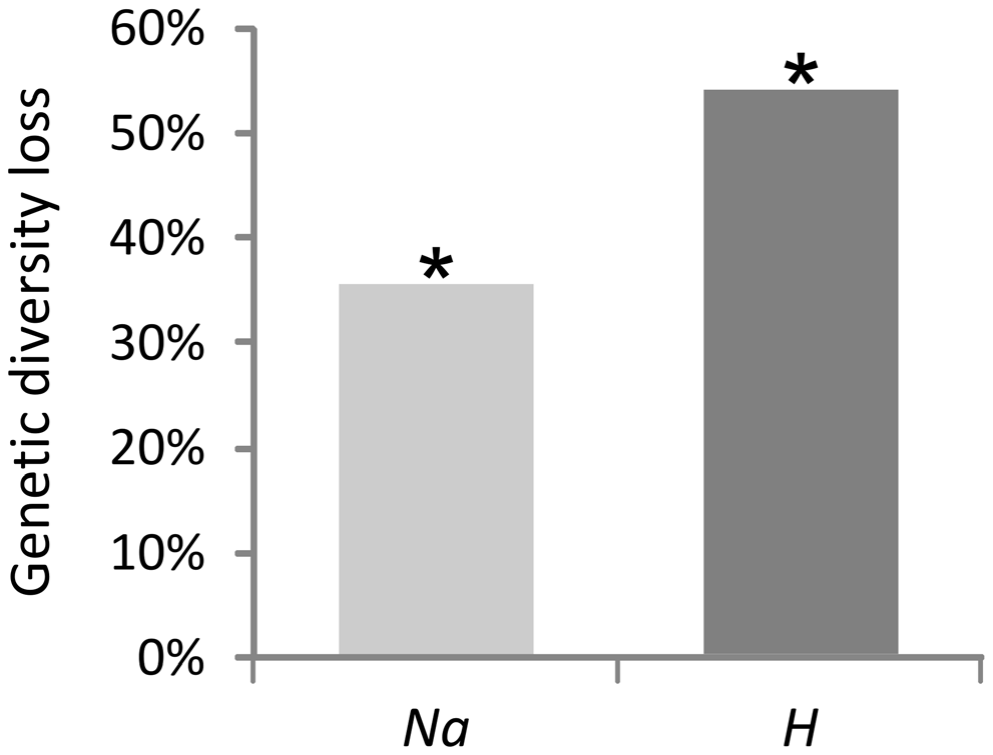
Loss of genetic diversity in the IAEA colony (2013 sample) with respect to its simulated source population at the Kenya/Uganda border in 1975. Na; allelic diversity loss and H; expected heterozygosity loss. Both losses are statistically significant (Wilcoson's signed-rank tests over loci, p≤0.05).

Posterior probability distributions of the effective population sizes (*Ne*, Figure 5) illustrate the intensity of genetic drift in the field populations and of the IAEA colony. *N_Bus_*. corresponds to Busia *Ne* before the bottleneck; the genetic data for the Busia sample did not contain any information about the Busia *Ne* after the genetic bottleneck (data not shown). All the posterior distributions of the *Ne* estimated substantially differ from the corresponding prior probability distributions (Figure 5) which indicates that the empirical data collected in Busia, Rukomeshi and the IAEA colony contain information about the estimated *Ne*. The estimated *Ne* for Busia was the smallest and similar to that for the IAEA colony. In contrast, the *Ne* estimated for Rukomeshi was an order of magnitude higher. A small effective population size was associated with the foundation and establishment of the IAEA colony and this genetic bottleneck was associated with an important loss of genetic diversity in the IAEA colony compared to its source population in 1975 (35.66% and 53.99% loss of *Na* and *H* respectively, p<0.05, 9 signed ranks, |W|≥35, Figure 6).

## Discussion

### The origin of the IAEA colony

The *G. pallidipes* IAEA colony is the only one available for mass rearing of this important vector of human and animal African trypanosomiasis. It is the source population for Sterile Insect Technique (SIT) programs in Kenya, Tanzania and Ethiopia [13]. In the present study we have analysed the genetic variation within and between the IAEA colony and its two potential source populations and combined it with historical information to identify the most likely source population and infer the past demography of the colony.

Measurements of genetic variation between samples and Bayesian clustering clearly indicated a strong similarity between the colony and the Busia population and no introgression from Rukomeshi. The only counter-evidence is four private alleles from Rukomeshi (present in Rukomeshi and not in Busia) that were observed in the IAEA colony. This result lacked statistical significance however. A potential explanation for this observation is that the Busia population experienced a genetic bottleneck between the establishment of the IAEA colony and the collection of our Busia sample. Those four alleles could have been lost in the Busia population through this genetic bottleneck which would imply that, while those four alleles are private to Rukomeshi relatively to our Busia sample, they might not have been private to Rukomeshi relatively to the Busia population before the genetic bottleneck.

The bottleneck in Busia is most likely to have been caused by tsetse control measures carried out in the area in the 1990's [70] and thus limit the utility of our Busia sample for drawing robust conclusions about the intensity of the genetic drift associated with the foundation and establishment of the IAEA colony in the 1970's through direct comparison of the genetic variation within samples. To overcome this restriction an ABC approach allowed inferences about the past demography of the IAEA colony based on contemporary samples of the colony and of its potential source population. The results suggest that taking into account the past demography of the source population was very relevant in the context of this study as it allowed us to make more accurate inferences about the intensity of the genetic drift that was associated with the foundation and establishment of the IAEA colony. Such inferences would have been biased in a direct comparison of the Busia sample and of the IAEA samples because the Busia population experienced a genetic bottleneck between the foundation of the IAEA colony and the sampling in Busia.

The ABC analysis gives a numerical prediction for the population size during the bottleneck. Our estimates revealed that the genetic drift associated with the foundation and establishment of the IAEA colony was strong with an effective number of individuals associated with the foundation and establishment of the colony around 27. This is consistent with the historical record that only 36 females were used to start the colony [14].

To conclude, all the analyses indicate clearly that the area of origin of the IAEA colony is the Kenya/Uganda border and that a severe genetic bottleneck was associated with the foundation and establishment of the colony causing a marked loss of genetic diversity. These data contrast with earlier findings on colonies of *G. pallidipes* but those studies were constrained by using isoenzyme markers and analytical approaches based largely on measurement of allele frequencies and heterozygosity [5], [74].

### Illustration of the usefulness of model based inferences in population genetics

The data set analysed in the present study is a good illustration of the usefulness of model based inferences, such as the ABC, over more classical population genetics analyses. It is clear from our data that a direct comparison of the genetic diversity of laboratory colony with that of the sample collected from its source population resulted in similar values for *Na* and AR which, in isolation, seem to indicate no loss of diversity in the colony. These findings contrast however with the heterozygosity excess, the mode shift of the distribution of the allele frequency classes and the ABC analysis all of which indicate that the Busia population has experienced a severe genetic bottleneck. Using the ABC it was possible to take into account the demography of the Busia population between the foundation of the IAEA colony and the sampling of flies in Busia. The current study clearly demonstrates that model based inferences, such as ABC, are more powerful at detecting genetic bottlenecks compared to moment based methods such as the ones implemented in the program Bottleneck [54] that was unable to detect any bottleneck in the IAEA colony from contemporary samples. This observation accords with previous findings both empirical [e.g. 75] and by simulation [76].

As indicated by Luikart et al. [57] and Piry et al. [54] the tests implemented in the program Bottleneck are able to detect recent genetic bottleneck 2*Ne*-4*Ne* generations before sampling. The bottleneck associated to the IAEA colony is likely to have occurred about 150 generations before sampling which potentially explains why the tests implemented in Bottleneck did not detect any bottleneck in the IAEA colony from contemporary samples. Moreover, Hoban et al. [77] recently showed by simulation that a “recovery after a moderate amount of time” is associated with an important reduction of power in bottleneck detection when using moment based methods. This finding is consistent with a rapid recovery after bottleneck that occurred in the IAEA colony from 36 to over 1500 females in approximately three years [14].

### Potential consequences for Sterile Insect Technique (SIT) programs

The high level of genetic drift that occurred in the IAEA colony could have a negative impact on SIT because some of the wild-type characteristics of *G. pallidipes* could potentially have been lost during the strong genetic bottleneck that was associated with the IAEA colony foundation and establishment. For example, some genetic diversity associated with mating competitiveness could have been lost that would lead to a suboptimal mating competitiveness of the colony males in the field.

Theoretically, several things can be done to reduce genetic drift associated with rearing organisms in captivity. In a colony already established it is possible to limit genetic drift by reducing the variation in reproductive success by reducing the number of non-mated individuals and/or by homogenising the contribution of each family to the next generation. However, for SIT, that requires mass production of males for sterilization and release into to field, reducing the variation in reproductive success is not feasible. Indeed, SIT requires the production of tens of thousands of insects and, in such conditions, the work load associated with measures seeking the homogenisation of the contribution of each family to the next generation would be too demanding. Moreover, for tsetse SIT, the requirement to produce spare males to be sterilised goes against any procedure that would aim at reducing the number of non-mated individuals. Indeed, males for SIT are spared before mating (and some techniques even seek to identify the males during the pupal period) and this is made possible by the use of a skewed sex ratio at mating [1 male to 3 or 4 females depending on tsetse species, 78].

For colony established from scratch, it is obvious that maximising the number of founder individuals and/or providing a continuous input of individuals from the field will limit the genetic diversity loss compared to the field population of origin. However, this could also prevent laboratory establishment and mass rearing that requires a high level of adaptation of the reared organisms to the laboratory conditions [4]. Indeed a continuous input of flies from the laboratory to the field would lead to a continuous gene flow from the wild to the laboratory and could prevent adaptation to the laboratory (i.e. the increase in frequency of “laboratory adapted” gene combinations).

In *G. pallidipes*, many unsuccessful attempts have been made to raise small laboratory colonies [e.g. 79] as well as larger mass rearing colonies [80]–[82]. Although some of those unsuccessful attempts could be due to fly diseases [82], genetic effects could also provide explanations. Surprisingly, compared to other *G. pallidipes* colonies reported in the literature, the IAEA colony was started from the smallest number of founding individuals while it is the only one that has reached mass rearing. Indeed, all the attempts to establish *G. pallidipes* colonies that are reported in the literature have been made with many more founding individuals [see 79, for failure, 79 & 83, for maintenance of very small colonies and 84 & 85 for successful colonisations]. The 36 producing females that were at the origin of the IAEA colony [14] may have carried a gene pool particularly suitable for laboratory colonisation. Alternatively it is possible that some deleterious alleles have been purged by genetic drift [86], [87], making this colony more successful than others.

Combining our results with those of Ouma et al. [35] allows evaluation of the representativeness of the IAEA colony compared to *G. pallidipes* field populations. When doing so it is important to take into account the fact that the Busia population likely experienced a genetic bottleneck, probably in the 1990's. In accordance with our inferences about this genetic bottleneck in Busia, Ouma et al. [35] have shown that the genetic diversity of the Busia population is on average around 25% (32% for *AR* and 21% for *H*) lower than that of other *G. pallidipes* populations in Uganda and Kenya. Taking into account this discrepancy of genetic diversity between Busia and other field populations, we can conclude that the genetic diversity of the IAEA colony is on average around 55% (45% for *AR* and 63% for *H*) lower than that of other *G. pallidipes* populations in Uganda and Kenya. This is concordant with our comparison between the genetic diversity of the IAEA colony with the simulated Busia 1975 sample (Figure 6). The differences between the genetic diversity present in the IAEA colony and populations that are genetically highly diverse like Rukomeshi [22] is of course even larger. Indeed, the genetic diversity in the IAEA colony is around 65% (65% for *AR* and 66% for *H*) lower than in the Rukomeshi population.

Insect laboratory colonies should be genetically similar to field populations for meaningful studies to be performed on different aspects of biology of a species [e.g. 5]. In this respect, our data imply that results obtained using the IAEA colony should be interpreted with some caution with regard to the biology of *G. pallidipes*. It should be noted that no genetic diversity loss compared to field populations have been detected in other colonies of different species of tsetse (*G. tachinoides*, *G. m. morsitans*, *G. m. centralis* and *G. pallidipes*) except in a *G. p. gambiensis* colony [reviewed in 5].

## Supporting Information

Figure S1 Successful amplification of locus GmmF10 in all the samples from Busia.
(PDF)

Supplementary file S1 Evaluation of 15 microsatellite loci for their use in population genetics analyses of *G. pallidipes* using multiplex PCR. Includes **Table S4** (*G. pallidipes* samples used in the evaluation of the 15 microsatellite loci), **Table S5** (Characteristics of the 15 microsatellites loci evaluated) and **Table S6** (Validation of the microsatellite loci for their use for population genetics).
(DOCX)

Supplementary file S2 Inferring the past demography of the Busia and the Rukomeshi populations using Approximate Bayesian computation (ABC). Includes **Table S7** (Prior distributions of the parameters used in the Busia ABC analysis) and **Figure S2** (Demographic scenario considered to infer the demographic history of the Busia and Rukomeshi populations).
(DOCX)

Table S1 Characteristics of the 9 microsatellites loci used in this study.
(DOCX)

Table S2 Allele “bins” defined for the 9 *G. pallidipes* microsatellite loci used.
(DOCX)

Table S3 Summary statistics used in the different ABC analyses.
(DOCX)
