# Supplementary material for: Laboratory Colonisation and Genetic Bottlenecks in the Tsetse Fly Glossina pallidipes
Source: PLoS Negl Trop Dis. 2014 Feb 13;8(2):e2697. doi: 10.1371/journal.pntd.0002697 (PMC3923722; doi:10.1371/journal.pntd.0002697)
Supplement: Figure S1 — Successful amplification of locus GmmF10 in all the samples from Busia. (PDF) [file pntd.0002697.s001.pdf]

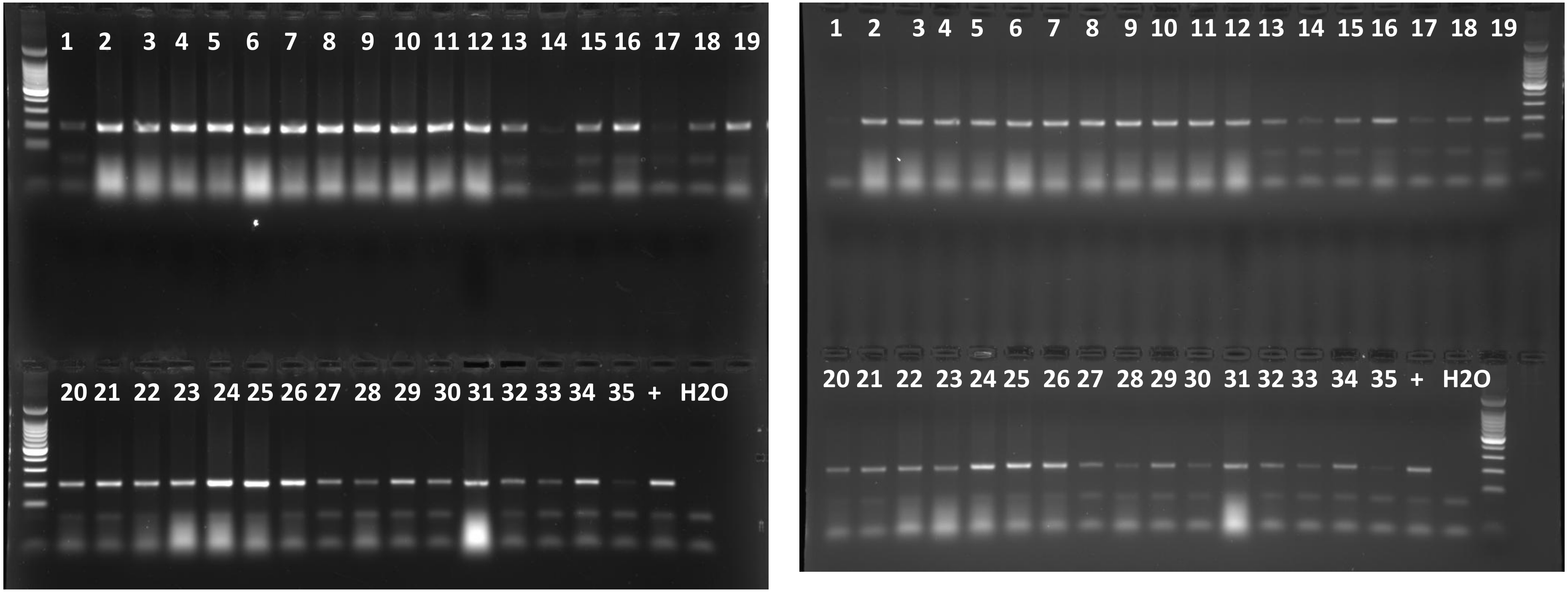

Figure S1:
Successful amplification of locus GmmF10 in all the samples from Busia. The pictures show the results of two independent PCRs.
