# Supplementary material for: Laboratory Colonisation and Genetic Bottlenecks in the Tsetse Fly Glossina pallidipes
Source: PLoS Negl Trop Dis. 2014 Feb 13;8(2):e2697. doi: 10.1371/journal.pntd.0002697 (PMC3923722; doi:10.1371/journal.pntd.0002697)
Supplement: Supplementary file S1 — Evaluation of 15 microsatellite loci for their use in population genetics analyses of G. pallidipes using multiplex PCR. Includes Table S4 (G. pallidipes samples used in the evaluation of the 15 microsatellite loci), Table S5 (Characteristics of the 15 microsatellites loci evaluated) and Table S6 (Validation of the microsatellite loci for their use for population genetics). (DOCX) [file pntd.0002697.s002.docx]

**Supplementary file S1: Evaluation of 15 microsatellite loci for their use in population genetics analyses of *G. pallidipes* using multiplex PCR**

**Sample collection and DNA extraction**

*G. pallidipes* were collected from two sites in Nguruman, Kenya (Sampu and Mukinyo) and two sites in the Shimba Hills National reserve, Kenya (Buffalo Ridge and Zungu Luka, Table S4). Trapping was performed using improved NGU2 traps [[1](#_ENREF_1)] baited with >3 weeks old cow urine and acetone. Tsetse were live caught and stored in 95% ethanol. DNA extractions were carried out from the abdomen of each fly using the Qiagen DNeasy blood and tissue kit following the manufacturer indications and using a final elution of 100µl.

**Table S4: *G. pallidipes* samples used in the evaluation of the 15 microsatellite loci**

| **Locality** | **Sites** | **Collection date** | **Latitude(°N), Longitude(°E)** | ***N_tot_*** | ***N_low_*** |
| --- | --- | --- | --- | --- | --- |
| Nguruman | Mukinyo | April 2011 | -1.8416, 36.0846 | 49 | 40 (GpA23b & GpC5b) |
|  | Sampu | April 2011 | -1.8916, 36.0689 | 30 | 27 (GpC5b) |
|  |  |  |  |  |  |
| Shimba Hills | Buffalo Ridge | May 2011 | -4.2425, 39.4372 | 35 | 23 (GpB20b) |
|  | Zungu Luka | Sept 2011 | -4.3387, 39.2638 | 36 | 31 (GpB20b) |

*N_tot_*: total number of individuals genotyped. *N_low_*: lower number of individuals for which genotypes were obtained (the loci corresponding to *N_low_* are indicated in brackets).

**Microsatellite genotyping**

15 polymorphic microsatellites loci previously described in the literature [[2-6](#_ENREF_2)] were evaluated for their use in population genetics analyses of *G. pallidipes* and multiplex PCR. Loci were combined into multiplex reactions with the help of Multiplex Manager v1.2 [[7](#_ENREF_7)] in an analysis of 2 millions iterations, a primer complementarity threshold of 7 and a minimum distance between loci of the same dye color of 26bp. The multiplex reactions were fine tuned by hand. These 15 loci, were amplified within three multiplex PCR as follow: reaction 1: GmmK06, GpA23b, GpC10b, GpC101, GpB115, GpCAG133; reaction 2: GmmA06, GmmK22, GpA19a, GmmB20, GpB20b, GpC26; reaction 3: GmmC17, GpB6b, GpC5b (Table S5). Multiplex PCR were carried out in a total volume of 10µl containing 2µl of template DNA solution, 1X Qiagen Multiplex PCR mix and 0.2µM of each primers. Forward PCR primers, 5’ labelled with a fluorescent dye, were used to allow the PCR products to be detected on an automated DNA sequencer. The PCR cycling conditions for both multiplex PCR were (95°C, 15min); 25 cycles of (94°C, 30s), (55°C, 90s) and (72°C, 60s); (60°C, 30min). 1µl of a 1/20 or 1/30 dilution of the multiplex PCR products were analysed by electrophoresis in combination with the GeneScan-500 LIZ size standard (Applied Biosystems) by DNA Sequencing & Services (MRCPPU, College of Life Sciences, University of Dundee, Scotland, www.dnaseq.co.uk) using Applied Biosystems Big-Dye Ver 3.1 chemistry on an Applied Biosystems model 3730 automated capillary DNA sequencer. The size estimation of amplified microsatellites was performed using GeneMarker v2.2.0 (SoftGenetics). The Excel Macro Autobin v0.9 [[8](#_ENREF_8)] was then used on the raw data set of amplified microsatellite sizes to automatically detect relevant gaps in size and thus help delimit allele “bins”. The allele “bins” defined using Autobin were then used within GeneMarker to automatically bin the alleles. Each peak was then manually checked.

**Validation of the microsatellite loci for their use for population genetics**

For each locus, the software Genepop on the Web [[9](#_ENREF_9),[10](#_ENREF_10)] was used to test for deviation from Hardy–Weinberg equilibrium with the probability test approach and to compute the Weir & Cockerham estimator of the coefficient of inbreeding *F*_IS_. The same software was used to test for linkage disequilibrium between loci. The significance levels of those later tests were corrected with Benjamini and Hochberg’s [[11](#_ENREF_11)] false discovery rate procedure when necessary as those tests can involve non orthogonal and multiple comparisons.

The complete dataset (150 individuals in 4 sites in Kenya) showed substantial polymorphism with an average of 11.333 alleles per locus over all samples. Over all samples, the number of alleles ranged from 3 at locus GmmC17 to 32 at locus GmmB20 (Table S5). Allele frequencies at three loci (GpA23b, GpB20b and GpB6b) were found to significantly deviate from Hardy-Weinberg expectations at more than one site and were excluded from the final multiplex set up (Table S6). These deviations from Hardy-Weinberg equilibrium were associated with heterozygote deficiency (positive *F*_IS_) and could be the results of the presence of null alleles at those loci. Significant linkage disequilibrium was detected between loci GpC5b and GpC101 (Table S6). An alignment of the DNA sequences corresponding to those two loci revealed that they actually correspond to a single locus (alignment not shown). We included GpC101 in the final multiplex set up because it was easier to combine with the other loci because of the fluorescent dye we associated to this locus and because it was slightly easier to score alleles at locus GpC101 than at locus GpC5b. Finally, loci GmmK22 and GmmB20 were excluded from the final multiplex set up because alleles were difficult to score at these loci (allele drop out and/or many stutter peaks, Table S6).

**Table S5: characteristics of the 15 microsatellites loci evaluated**

| **multiplex reaction** | **Locus** | **repeat motif** | **Seq. ID** | **Primer sequences 5'-3** | **Dye on F primer (supplier)** | **allele size range^#^ (bp)** | **Number of alleles^#^** | **Original reference** |
| --- | --- | --- | --- | --- | --- | --- | --- | --- |
| 1 | GmmK06 | (ATGT)_12_* | Tfly_23-t504k06.q1k | F: TAACGTGCATGTGCGTGTG R: CCATCAATACGAGCAGACCG | 6-FAM (Biomers.net) | 119-131 | 5 | [[3](#_ENREF_3),[4](#_ENREF_4)] |
| 1 | GpA23b | (GT)_14_ | AY220499 | F: CTCCTGCTTGGGCTCTAT R: GCGATGAGTTGGTTTCTTT | 6-FAM (Biomers.net) | 175-219 | 16 | [[5](#_ENREF_5)] |
| 1 | GpC10b | (CAT)_9_ | AY220503 | F: GTTGATGTTGTGATGGTAATGA R: GCTGGCAAAGAAACTAATGA | 6-FAM (Biomers.net) | 291-309 | 7 | [[5](#_ENREF_5)] |
| 1 | GpC101 | (TGA)_11_ | DQ168824 | F: CCTCAATACAGCAGCAGATG R: CAAGGTGTGTTGTCGTCTTC | HEX (Biomers.net) | 201-221 | 14 | [[6](#_ENREF_6)] |
| 1 | GpB115 | (CT)_16_ | DQ168823 | F: AGCGATAGAAAGGGTCAATC R: CGTAGAGATAGCGAGAGTGTG | NED (Applied Biosystems) | 144-164 | 10 | [[6](#_ENREF_6)] |
| 1 | GpCAG133 | (CAA)_6_** | AY033512 | F: ATTTTTGCGTCAACGTGA R: ATGAGGATGTTGTCCAGTTT | PET (Applied Biosystems) | 185-197 | 5 | [[2](#_ENREF_2),[12](#_ENREF_12)] |
|  |  |  |  |  |  |  |  |  |
| 2 | GmmA06 | (CA)_16_* | Tfly_23-t487a06.q1k | F: ACTTCCATGTTATGTTCGTTGC R: TGCCTTAGTTGAGAAACTCTGC | 6-FAM (Biomers.net) | 154-186 | 12 | [[3](#_ENREF_3),[4](#_ENREF_4)] |
| 2 | GmmK22 | (TGT)_20_* | Tfly_23-t513k22.p1k | F: ACGCTTACGTTTCCGTTACAC R: AAGCTAACCGAACCAGCAC | 6-FAM (Biomers.net) | 186-204 | 5 | [[3](#_ENREF_3),[4](#_ENREF_4)] |
| 2 | GpA19a | (CA)_7_GA(CA)_8_ | AY220498 | F: CATATCCACACCCACATACAT R: GCGATTATGGCTAGAGGTTT | HEX (Biomers.net) | 138-172 | 7 | [[5](#_ENREF_5)] |
| 2 | GmmB20 | (GT)_33_* | Tfly_23-t572b20.q1k | F: AAATGCATGTCTAACTGTCCG R: AGCAAAAGGCAACTAAAGTGATG | HEX (Biomers.net) | 186-262 | 32 | [[3](#_ENREF_3),[4](#_ENREF_4)] |
| 2 | GpB20b | (GA)_30_ | AY220501 | F: AGTTTGCTTCTCAACGCAGTAG R: TTCGGCAGTAGATGGCAA | NED (Applied Biosystems) | 149-187 | 18 | [[5](#_ENREF_5)] |
| 2 | GpC26b | (CAT)_3_CGT(CAT)_12_ | AY220504 | F: GGATCACCCTTCTTGAATG R: GGACGTTATTTGTTCGTGTAA | PET (Applied Biosystems) | 172-201 | 10 | [[5](#_ENREF_5)] |
|  |  |  |  |  |  |  |  |  |
| 3 | GmmC17 | (TA)_5_(TGTA)_13_(GT)_7_* | Tfly_23-t506c17.p1k | F: TGCGCTTTGAACGGAACG R: CTATGCCGCCTGGCTTATC | 6-FAM (Biomers.net) | 190-202 | 3 | [[3](#_ENREF_3),[4](#_ENREF_4)] |
| 3 | GpB6b | (CT)_15_ | AY220500 | F: GTAAACCGCCTGTCACATC R: AGGGAGAGAGCCGTAAGAG | HEX (Biomers.net) | 187-217 | 12 | [[5](#_ENREF_5)] |
| 3 | GpC5b | (TGA)_11_ | AY220502 | F: GTTGTTTTCTGCTCCTCAATA R: CAAGGGTGTGTCGTCTTC | NED (Applied Biosystems) | 212-232 | 14 | [[5](#_ENREF_5)] |

Seq. ID: Accession number or *Glossina morsitans morsitans* genome sequence ID. * in *Glossina morsitans morsitans* genome. **: the microsatellite repeat is more complex than (CAA)_6_. The microsatellite is made of intercalation of CAA and CAG motifs. In the published sequence (A7033512), the microsatellite region includes 9 CAG motifs and 16 CAA motifs. #: when considering the four sites in Nguruman and Shimba Hills National Reserve.

**Table S6: Validation of the microsatellite loci for their use for population genetics**

|  |  | **Nguruman** | | | | |  | **Shimba Hills** | | | | | **reason for exclusion from the final multiplex set up** |
| --- | --- | --- | --- | --- | --- | --- | --- | --- | --- | --- | --- | --- | --- |
|  |  | **Mukinyo** | |  | **Sampu** | |  | **Buffalo Ridge** | |  | **Zungu Luka** | |  |
| **multiplex reaction** | **Locus** | ***F*_IS_** | **HWE** |  | ***F*_IS_** | **HWE** |  | ***F*_IS_** | **HWE** |  | ***F*_IS_** | **HWE** |  |
| 1 | GmmK06 | - | - |  | - | - |  | **0.438** | **0.012** |  | 0.094 | 0.593 |  |
|  | GpA23b | **0.817** | **0** |  | **0.752** | **0** |  | **0.355** | **0.002** |  | **0.396** | **0** | deviation from HWE in more than one site |
|  | GpC10b | 0.060 | 0.863 |  | 0.076 | 0.488 |  | -0.118 | 0.627 |  | -0.141 | 0.201 |  |
|  | GpC101 | -0.178 | 0.352 |  | 0.059 | 0.955 |  | 0.028 | 0.055 |  | **0.107** | **0.040** |  |
|  | GpB115 | 0.071 | 0.906 |  | 0.082 | 0.338 |  | -0.041 | 0.253 |  | -0.108 | 0.835 |  |
|  | GpCAG133 | -0.011 | 0.109 |  | 0.038 | 0.481 |  | 0.014 | 1 |  | -0.137 | 0.830 |  |
| 2 | GmmA06 | 0.009 | 0.499 |  | 0.166 | 0.285 |  | -0.017 | 0.802 |  | -0.009 | 0.831 |  |
|  | GmmK22 | 0.164 | 0.376 |  | 0.081 | 0.711 |  | 0.231 | 0.348 |  | 0.132 | 0.236 | allele drop out can be strong |
|  | GpA19a | -0.098 | 0.394 |  | -0.045 | 0.117 |  | 0.142 | 0.677 |  | -0.084 | 0.392 |  |
|  | GmmB20 | -0.051 | 0.706 |  | 0.090 | 0.139 |  | 0.065 | 0.306 |  | 0.056 | 0.205 | allele drop out can be strong, many stutter peaks |
|  | GpB20b | -0.109 | 0.073 |  | -0.024 | 0.351 |  | **0.36** | **0.001** |  | **0.393** | **0** | deviation from HWE in more than one site |
|  | GpC26b | 0.050 | 0.686 |  | -0.043 | 0.507 |  | 0.138 | 0.320 |  | 0.031 | 0.284 |  |
| 3 | GmmC17 | 0.073 | 0.683 |  | 0.017 | 1 |  | 0.013 | 0.728 |  | -0.178 | 0.205 |  |
|  | GpB6b | 0.004 | 0.775 |  | 0.053 | 0.116 |  | **0.457** | **0** |  | **0.483** | **0** | deviation from HWE in more than one site |
|  | GpC5b | -0.038 | 0.476 |  | 0.099 | 0.722 |  | 0.009 | 0.214 |  | **0.121** | **0.046** | linkage desequilibrium with with GpC101 (p<0.001) |

HWE: Pvalue of the probability test of deviation from Hardy-Weinberg equilibrium

**References**

1. Brightwell R, Dransfield RD, Kyorku C (1991) Development of a low-cost tsetse trap and odor baits for *Glossina pallidipes* and *G. longipennis* in Kenya. Med Vet Entomol 5: 153-164.

2. Baker MD, Krafsur ES (2001) Identification and properties of microsatellite markers in tsetse flies *Glossina morsitans* sensu lato (Diptera: Glossinidae). Mol Ecol Notes 1: 234-236.

3. Hyseni C, Beadell JS, Gomez Ocampo Z, Ouma JO, Okedi LM, et al. (2011) The *G.m. morsitans* (Diptera: Glossinidae) genome as a source of microsatellite markers for other tsetse fly (Glossina) species. Molecular Ecology Resources Primer Database, Available at: <http://tomatobioltrinityedu/manuscripts/11-3/mer-10-0402pdf> [Accessed May 2013].

4. Molecular Ecology Resources Primer Development C, Agata K, Alasaad S, Almeida-Val VMF, ÁLvarez-Dios JA, et al. (2011) Permanent Genetic Resources added to Molecular Ecology Resources Database 1 December 2010–31 January 2011. Mol Ecol Resources 11: 586-589.

5. Ouma JO, Cummings MA, Jones KC, Krafsur ES (2003) Characterization of microsatellite markers in the tsetse fly, *Glossina pallidipes* (Diptera : Glossinidae). Mol Ecol Notes 3: 450-453.

6. Ouma JO, Marquez JG, Krafsur ES (2006) New polymorphic microsatellites in *Glossina pallidipes* (Diptera : Glossinidae) and their cross-amplification in other tsetse fly taxa. Biochem Genet 44: 471-477.

7. Holleley CE, Geerts PG (2009) Multiplex Manager 1.0: a cross-platform computer program that plans and optimizes multiplex PCR. Biotechniques 46: 511-517.

8. Salin F AutoBin, Available at: www4.bordeaux-aquitaine.inra.fr/biogeco/Media/Ressources/Logiciels/Autobin [Accessed July 2013]. v 0.9 ed: INRA. pp. AutoBin is Excel Macro written in Microsoft Visual Basic (VBA) . It automatically analyzes raw data generated from microsatellites genotyping software such as STRand (Toonen et al., 2001) or GeneMapper (Applied Biosystems, USA). It can deal with unlimited number of loci and samples, with no consideration of the type of SSRs motifs. AutoBin helps the user to bin his data with visual alerts and format automatically the data for downstream analysis.

9. Raymond M, Rousset F (1995) Genepop (version. 1.2), a population genetics software for exact tests and ecumenicism. J Hered 86: 248-249.

10. Rousset F (2008) GENEPOP ' 007: a complete re-implementation of the GENEPOP software for Windows and Linux. Mol Ecol Resources 8: 103-106.

11. Benjamini Y, Hochberg Y (1995) Controlling the False Discovery Rate - a Practical and Powerful Approach to Multiple Testing. J Roy Stat Soc B Met 57: 289-300.

12. Krafsur ES (2002) Population structure of the tsetse fly *Glossina pallidipes* estimated by allozyme, microsatellite and mitochondrial gene diversities. Insect Molecular Biology 11: 37-45.
