# Supplementary material for: Laboratory Colonisation and Genetic Bottlenecks in the Tsetse Fly Glossina pallidipes
Source: PLoS Negl Trop Dis. 2014 Feb 13;8(2):e2697. doi: 10.1371/journal.pntd.0002697 (PMC3923722; doi:10.1371/journal.pntd.0002697)
Supplement: Supplementary file S2 — Inferring the past demography of the Busia and the Rukomeshi populations using Approximate Bayesian computation (ABC). Includes Table S7 (Prior distributions of the parameters used in the Busia ABC analysis) and Figure S2 (Demographic scenario considered to infer the demographic history of the Busia and Rukomeshi populations). (DOCX) [file pntd.0002697.s003.docx]

**Supplementary file S2. Inferring the past demography of the Busia and the Rukomeshi populations using Approximate Bayesian computation (ABC)**

Analysis 1 was focused on Busia and analysis 2 on Rukomeshi. In each of these analyses we compared three scenarios (Figure S2). The prior distributions of the parameters used in this analysis are indicated in Table S7.

In ABC analysis 1 we compared the following three scenarios: (A) Busia population experienced a strong population bottleneck, starting in 1991, with a small effective population size *NB*_cont.Bus._ and a recovery to a large effective population size *N*_Bus._ before 2000 (Figure S2A),. We believe this scenario is likely to represent the decrease of tsetse apparent density >95% that was recorded in the area following the tsetse control that started in 1991 [[1](#_ENREF_1)] (B) the Busia population experienced a lighter reduction in population size between 1975 and 1985 from a large effective population size *N*_Bus._ to a smaller effective population size *N_destruc.Bus_*_._ (Figure S2B). Here we assumed that the reduction in population size was the consequence of tsetse habitat destruction associated with the increase of the human population in the area which was the most important between 1975 and 1985 (<http://www.citypopulation.de/php/kenya-admin.php?adm2id=40>). In that scenario, *N*_Bus._ and *N_destruc.Bus_*_._ were drawn from the same prior distributions (Table S7) but *N_destruc.Bus_*_._ was forced to be smaller to *N*_Bus._ (C) the effective population size *N*_Bus._ remains constant (Figure S2C).

In ABC analysis 2 we compared the following three scenarios similarly to what was done for Busia: (A) Rukomeshi population experienced a strong population bottleneck, starting in 1991 and that could last up to 25 generations (*BD*_cont.Ruk._, Table S7). We assumed that the population bottleneck in Rukomeshi would have been shorter in Rukomeshi than in Busia (up to 47 generations) because the impact of the field trial from Hargrove and Langley [[2](#_ENREF_2)] is likely to have had a more limited impact on the *G. pallidipes* population size than a full-scale tsetse control programme. In the first model the bottleneck was associated with a small effective population size *NB*_cont.Ruk._ and a recovery to a large effective population size *N_Ruk._* after *BD*_cont.Ruk._ generations. (B) the Rukomeshi population experienced a lighter reduction in population size after the field trial [[2](#_ENREF_2)] from a large effective population size *N_Ruk._* to a smaller effective population size *N_light.Ruk_*_._. *N_Ruk._* and *N_light.Ruk_*_._ were drawn from the same prior distributions (Table S7) but *N_light.Ruk_*_._ was forced to be smaller to *N*_Bus._ (C) the effective population size *N_Ruk._* remains constant.

**Table S7: Prior distributions of the parameters used in the Busia and Rukomeshi ABC analyses**

| **Generic names of the parameters in figure 1** | **Parameters** | **Prior distribution** |
| --- | --- | --- |
| *N*_i_ | *N*_Bus._, *N_Ruk._* | Uniform [500; 20000] |
| *N*_j_ | *N_destruc.Bus_*_._, *N_light.Ruk_*_._ | Uniform [500; 20000]  With *N*_Bus._ > *N_destruc.Bus_*_._ & *N_Ruk._* > *N_light.Ruk_*_._ |
| *NB*_i_ | *NB*_cont.Bus._*, NB*_cont.Ruk._ | Uniform [1; 100] |
| *T_i_* | *T_destruc.Bus._* | Uniform [1975; 1985] |
|  | *T_Ruk._* | 1991 |
| *BD*_i_ | *BD*_cont.Bus._ | Uniform [1, 47] |
|  | *BD*_cont.Ruk._ | Uniform [1, 25] |
| - | Mutational model | GSM+SNI with default parameters |
| - | Number of generations per year | 5* |

**Note:** *N*_Bus._ & *N_Ruk._*: respectively the effective size of the Busia and Rukomeshi population. *NB*_cont.Bus._& *NB*_cont.Ruk._: respectively the effective size of the Busia and the Rukomeshi population during the population bottleneck in scenario 1. *N_destruc.Bus_*_._& *N_light.Ruk_*_._: respectively the effective size of Busia and Rukomeshi population after the reduction of population size in scenario 2. *T_destruc.Bus._*: date at which the reduction of Busia population size starts in scenario 2. *T_Ruk._*: date at which the reduction of Rukomeshi population size starts in scenario 2. *BD*_cont._ & *BD*_cont.Ruk._: duration of the population bottleneck in scenario 1 for Busia and Rukomeshi respectively. GSM: generalized stepwise mutation model. SNI: single locus indel mutations. *: Assuming an average optimal age at mating of 10 days [[3](#_ENREF_3)], an average duration of the pupal period of 35 days [[4](#_ENREF_4)] and an average production of 3 pupae per female [[5](#_ENREF_5)] we estimated the average generation time to 75 days and thus performed our ABC analyses assuming an average of 5 generations per year.

| 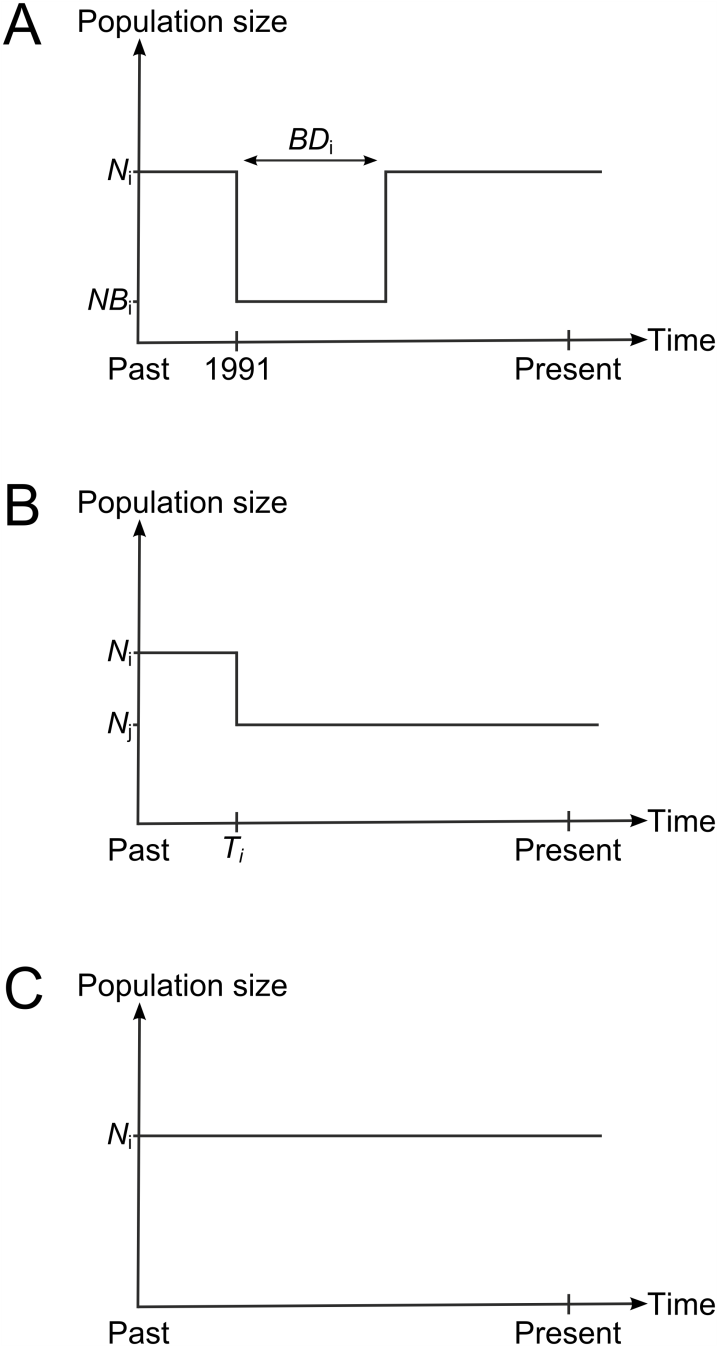 | **Figure S2: Demographic scenario considered to infer the demographic history of the Busia and Rukomeshi populations.** A, B and C: Demographic diagrams of scenarios 1, 2 and 3 respectively. *N_i_*: stable effective size of the population. *NB*_i_: effective size of the population during the population bottleneck. *BD*_i_: duration of the population bottleneck. *N_j_*: effective size of the population after the reduction of population size in scenario 2. *T_i_*: date at which the reduction of population size starts in scenario 2. |
| --- | --- |

**References**

1. Magona JW, Okuna NM, Katabazi BK, Omollo P, Okoth JO, et al. (1998) Control of tsetse and animal trypanosomosis using a combination of tsetse-trapping, pour-on and chemotherapy along the Uganda-Kenya border. Rev Elev Med Vet Pay 51: 311-315.

2. Hargrove JW, Langley PA (1993) A field trial of pyriproxyfen-treated targets as an alternative method for controlling tsetse (Diptera, Glossinidae). B Entomol Res 83: 361-368.

3. Olet PA, Opiyo E, Robinson AS (2002) Sexual receptivity and age in *Glossina pallidipes* Austen (Dipt., Glossinidae). J Appl Entomol 126: 86-91.

4. Boucias DG, Kariithi HM, Bourtzis K, Schneider DI, Kelley K, et al. (2013) Transgenerational transmission of the *Glossina pallidipes* hytrosavirus depends on the presence of a punctional symbiome. Plos One 8: e61150.

5. Abd-Alla AMM, Adun H, Parker AG, Vreysen MJB, Bergoin M (2012) The Antiviral Drug Valacyclovir Successfully Suppresses Salivary Gland Hypertrophy Virus (SGHV) in Laboratory Colonies of *Glossina pallidipes*. Plos One 7: e38417.
