# Supplementary material for: Laboratory Colonisation and Genetic Bottlenecks in the Tsetse Fly Glossina pallidipes
Source: PLoS Negl Trop Dis. 2014 Feb 13;8(2):e2697. doi: 10.1371/journal.pntd.0002697 (PMC3923722; doi:10.1371/journal.pntd.0002697)
Supplement: Table S1 — Characteristics of the 9 microsatellites loci used in this study. (DOCX) [file pntd.0002697.s004.docx]

**Table S1: characteristics of the 9 microsatellites loci used in this study.**

| **multiplex reaction** | **Locus** | **repeat motif** | **Seq. ID** | **Primer sequences 5'-3** | **Dye on F primer (supplier)** | **allele size range^#^ (bp)** | **Number of alleles^#^** | **Original reference** |
| --- | --- | --- | --- | --- | --- | --- | --- | --- |
| α | GmmK06 | (ATGT)_12_* | Tfly_23-t504k06.q1k | F: TAACGTGCATGTGCGTGTG  R: CCATCAATACGAGCAGACCG | 6-FAM (Biomers.net) | 117-129 | 5 | [[1](#_ENREF_1),[2](#_ENREF_2)] |
| α | GmmC17 | (TA)_5_(TGTA)_13_(GT)_7_* | Tfly_23-t506c17.p1k | F: TGCGCTTTGAACGGAACG  R: CTATGCCGCCTGGCTTATC | 6-FAM (Biomers.net) | 187-201 | 4 | [[1](#_ENREF_1),[2](#_ENREF_2)] |
| α | GpC10b | (CAT)_9_ | AY220503 | F: GTTGATGTTGTGATGGTAATGA  R: GCTGGCAAAGAAACTAATGA | 6-FAM (Biomers.net) | 291-309 | 6 | [[3](#_ENREF_3)] |
| α | GpC101 | (TGA)_11_ | DQ168824 | F: CCTCAATACAGCAGCAGATG  R: CAAGGTGTGTTGTCGTCTTC | HEX (Biomers.net) | 200-220 | 13 | [[4](#_ENREF_4)] |
| α | GpB115 | (CT)_16_ | DQ168823 | F: AGCGATAGAAAGGGTCAATC  R: CGTAGAGATAGCGAGAGTGTG | NED (Applied Biosystems) | 147-163 | 9 | [[4](#_ENREF_4)] |
| α | GpCAG133 | (CAA)_6_** | AY033512 | F: ATTTTTGCGTCAACGTGA  R: ATGAGGATGTTGTCCAGTTT | PET (Applied Biosystems) | 184-196 | 5 | [[5](#_ENREF_5),[6](#_ENREF_6)] |
|  |  |  |  |  |  |  |  |  |
| β | GmmA06 | (CA)_16_ * | Tfly_23-t487a06.q1k | F: ACTTCCATGTTATGTTCGTTGC  R: TGCCTTAGTTGAGAAACTCTGC | 6-FAM (Biomers.net) | 153-175 | 12 | [[1](#_ENREF_1),[2](#_ENREF_2)] |
| β | GpA19a | (CA)_7_GA(CA)_8_ | AY220498 | F: CATATCCACACCCACATACAT  R: GCGATTATGGCTAGAGGTTT | HEX (Biomers.net) | 137-171 | 8 | [[3](#_ENREF_3)] |
| β | GpC26b | (CAT)_3_CGT(CAT)_12_ | AY220504 | F: GGATCACCCTTCTTGAATG  R: GGACGTTATTTGTTCGTGTAA | PET (Applied Biosystems) | 171-198 | 9 | [[3](#_ENREF_3)] |

Seq. ID: Accession number or *Glossina morsitans morsitans* genome sequence ID. * in *Glossina morsitans morsitans* genome. **: the microsatellite repeat is more complex than (CAA)_6_and is made of intercalation of CAA and CAG motifs. In the published sequence (A7033512), the microsatellite region includes 9 CAG motifs and 16 CAA motifs. #: when considering Rukomeshi, Busia and the IAEA colony.

**References:**

1. Hyseni C, Beadell JS, Gomez Ocampo Z, Ouma JO, Okedi LM, et al. (2011) The *G.m. morsitans* (Diptera: Glossinidae) genome as a source of microsatellite markers for other tsetse fly (Glossina) species. Molecular Ecology Resources Primer Database, Available at: <http://tomatobioltrinityedu/manuscripts/11-3/mer-10-0402pdf> [Accessed May 2013].

2. Molecular Ecology Resources Primer Development C, Agata K, Alasaad S, Almeida-Val VMF, ÁLvarez-Dios JA, et al. (2011) Permanent Genetic Resources added to Molecular Ecology Resources Database 1 December 2010–31 January 2011. MOL ECOL RESOUR 11: 586-589.

3. Ouma JO, Cummings MA, Jones KC, Krafsur ES (2003) Characterization of microsatellite markers in the tsetse fly, *Glossina pallidipes* (Diptera : Glossinidae). Mol Ecol Notes 3: 450-453.

4. Ouma JO, Marquez JG, Krafsur ES (2006) New polymorphic microsatellites in *Glossina pallidipes* (Diptera : Glossinidae) and their cross-amplification in other tsetse fly taxa. Biochem Genet 44: 471-477.

5. Baker MD, Krafsur ES (2001) Identification and properties of microsatellite markers in tsetse flies *Glossina morsitans* sensu lato (Diptera: Glossinidae). Mol Ecol Notes 1: 234-236.

6. Krafsur ES (2002) Population structure of the tsetse fly *Glossina pallidipes* estimated by allozyme, microsatellite and mitochondrial gene diversities. Insect Molecular Biology 11: 37-45.
