# Supplementary material for: Laboratory Colonisation and Genetic Bottlenecks in the Tsetse Fly Glossina pallidipes
Source: PLoS Negl Trop Dis. 2014 Feb 13;8(2):e2697. doi: 10.1371/journal.pntd.0002697 (PMC3923722; doi:10.1371/journal.pntd.0002697)
Supplement: Table S2 — Allele “bins” defined for the 9 G. pallidipes microsatellite loci used. (DOCX) [file pntd.0002697.s005.docx]

**Table S2: Allele “bins” defined for the 9 *G. pallidipes* microsatellite loci used**

| **multiplex reaction** | **Locus** | **Allele name** | **Bin size (bp)** | **Lower limit of the bin (bp)** | **Higher limit of the bin (bp)** |
| --- | --- | --- | --- | --- | --- |
| α | GmmK06 | 117 | 117.10 | 0.5 | 0.5 |
|  |  | 119 | 119.30 | 0.5 | 0.5 |
|  |  | 125 | 125.40 | 0.5 | 0.5 |
|  |  | 127 | 127.60 | 0.5 | 0.5 |
|  |  | 129 | 129.40 | 0.5 | 0.5 |
|  |  |  |  |  |  |
| α | GmmC17 | 187 | 187.20 | 0.5 | 0.5 |
|  |  | 189 | 189.30 | 0.6 | 0.5 |
|  |  | 191 | 191.60 | 0.5 | 0.5 |
|  |  | 201 | 201.00 | 0.5 | 0.5 |
|  |  |  |  |  |  |
| α | GpC10b | 291 | 290.70 | 0.7 | 0.8 |
|  |  | 297 | 297.00 | 0.5 | 0.5 |
|  |  | 300 | 300.50 | 0.6 | 0.6 |
|  |  | 303 | 303.10 | 0.7 | 0.8 |
|  |  | 306 | 306.60 | 0.5 | 0.6 |
|  |  | 309 | 310.00 | 0.5 | 0.5 |
|  |  |  |  |  |  |
| α | GpC101 | 200 | 200.40 | 0.5 | 0.5 |
|  |  | 202 | 202.00 | 0.4 | 0.4 |
|  |  | 203 | 203.10 | 0.4 | 0.4 |
|  |  | 205 | 205.20 | 0.4 | 0.4 |
|  |  | 206 | 206.40 | 0.4 | 0.4 |
|  |  | 207 | 207.70 | 0.6 | 0.6 |
|  |  | 209 | 209.40 | 0.5 | 0.5 |
|  |  | 212 | 212.40 | 0.5 | 0.5 |
|  |  | 214 | 214.00 | 0.5 | 0.5 |
|  |  | 215 | 215.40 | 0.5 | 0.5 |
|  |  | 217 | 217.00 | 0.4 | 0.4 |
|  |  | 218 | 218.10 | 0.4 | 0.4 |
|  |  | 220 | 220.20 | 0.5 | 0.5 |
|  |  |  |  |  |  |
| α | GpB115 | 147 | 146.10 | 0.5 | 0.5 |
|  |  | 149 | 148.40 | 0.5 | 0.5 |
|  |  | 151 | 150.80 | 0.6 | 0.5 |
|  |  | 153 | 152.80 | 0.5 | 0.5 |
|  |  | 155 | 154.90 | 0.5 | 0.5 |
|  |  | 157 | 157.00 | 0.5 | 0.5 |
|  |  | 159 | 159.00 | 0.5 | 0.5 |
| α | GpB115 | 161 | 161.30 | 0.6 | 0.6 |
|  |  | 163 | 163.00 | 0.5 | 0.5 |
|  |  |  |  |  |  |
| α | GpCAG133 | 184 | 184.60 | 0.5 | 0.5 |
|  |  | 187 | 187.70 | 0.5 | 0.5 |
|  |  | 190 | 190.50 | 0.5 | 0.5 |
|  |  | 193 | 193.10 | 0.6 | 0.6 |
|  |  | 196 | 196.30 | 0.5 | 0.5 |
|  |  |  |  |  |  |
| β | GmmA06 | 153 | 153.70 | 0.5 | 0.5 |
|  |  | 155 | 155.40 | 0.5 | 0.5 |
|  |  | 157 | 157.30 | 0.5 | 0.5 |
|  |  | 159 | 159.10 | 0.5 | 0.5 |
|  |  | 161 | 161.26 | 0.5 | 0.5 |
|  |  | 163 | 163.00 | 0.5 | 0.5 |
|  |  | 165 | 165.20 | 0.6 | 0.5 |
|  |  | 167 | 167.40 | 0.5 | 0.5 |
|  |  | 169 | 168.80 | 0.5 | 0.5 |
|  |  | 171 | 170.80 | 0.5 | 0.5 |
|  |  | 173 | 172.70 | 0.5 | 0.5 |
|  |  | 175 | 174.80 | 0.5 | 0.5 |
|  |  |  |  |  |  |
| β | GpA19a | 137 | 137.20 | 0.5 | 0.5 |
|  |  | 147 | 146.90 | 0.5 | 0.5 |
|  |  | 149 | 149.00 | 0.5 | 0.5 |
|  |  | 153 | 154.00 | 0.5 | 0.5 |
|  |  | 155 | 156.00 | 0.4 | 0.4 |
|  |  | 157 | 158.00 | 0.4 | 0.4 |
|  |  | 159 | 159.80 | 0.5 | 0.5 |
|  |  | 171 | 171.70 | 0.5 | 0.5 |
|  |  |  |  |  |  |
| β | GpC26b | 171 | 171.50 | 0.5 | 0.5 |
|  |  | 174 | 174.30 | 0.5 | 0.5 |
|  |  | 177 | 177.40 | 0.5 | 0.5 |
|  |  | 180 | 180.10 | 0.6 | 0.7 |
|  |  | 183 | 183.00 | 0.5 | 0.5 |
|  |  | 186 | 185.90 | 0.5 | 0.5 |
|  |  | 187 | 187.10 | 0.4 | 0.4 |
|  |  | 195 | 194.60 | 0.5 | 0.5 |
|  |  | 198 | 197.20 | 0.5 | 0.5 |
