# Supplementary material for: Laboratory Colonisation and Genetic Bottlenecks in the Tsetse Fly Glossina pallidipes
Source: PLoS Negl Trop Dis. 2014 Feb 13;8(2):e2697. doi: 10.1371/journal.pntd.0002697 (PMC3923722; doi:10.1371/journal.pntd.0002697)
Supplement: Table S3 — Summary statistics used in the different ABC analyses. (DOCX) [file pntd.0002697.s006.docx]

**Table S3: summary statistics used in the different ABC analyses**

| **Analysis** | **number** | **Summary statistics** |
| --- | --- | --- |
| **1** | **1** | *Na* in Busia sample |
|  | **2** | *H* in Busia sample |
|  | **3** | *VAR* in Busia sample |
|  | **4** | MGW in Busia sample |
|  |  |  |
| **2** | **1** | *Na* in Rukomeshi sample |
|  | **2** | *H* in Rukomeshi sample |
|  | **3** | *var* in Rukomeshi sample |
|  | **4** | MGW in Rukomeshi sample |
|  |  |  |
| **3** | **1** | *Na* in Busia sample |
|  | **2** | *Na* in IAEA2012 sample |
|  | **3** | *Na* in IAEA2013 sample |
|  | **4** | *Na* in Rukomeshi sample |
|  | **5** | *H* in Busia sample |
|  | **6** | *H* in IAEA2012 sample |
|  | **7** | *H* in IAEA2013 sample |
|  | **8** | *H* in Rukomeshi sample |
|  | **9** | *var* in Busia sample |
|  | **10** | *var* in IAEA2012 sample |
|  | **11** | *var* in IAEA2013 sample |
|  | **12** | *var* in Rukomeshi sample |
|  | **13** | MGW in Busia sample |
|  | **14** | MGW in IAEA2012 sample |
|  | **15** | MGW in IAEA2013 sample |
|  | **16** | MGW in Rukomeshi sample |
|  | **17** | pairwise *F*_ST_ Busia-IAEA2012 |
|  | **18** | pairwise *F*_ST_ Busia-IAEA2013 |
|  | **19** | pairwise *F*_ST_ Busia- Rukomeshi |
|  | **20** | pairwise *F*_ST_ IAEA2012- IAEA2013 |
|  | **21** | pairwise *F*_ST_ IAEA2012- Rukomeshi |
|  | **22** | pairwise *F*_ST_ IAEA2013- Rukomeshi |
|  | **23** | *L*_i🡪j_ Busia 🡪 IAEA2012 |
|  | **24** | *L*_i🡪j_ IAEA2012 🡪 Busia |
|  | **25** | *L*_i🡪j_ Busia 🡪 IAEA2013 |
|  | **26** | *L*_i🡪j_ IAEA2013 🡪 Busia |
|  | **27** | *L*_i🡪j_ Busia 🡪 Rukomeshi |
|  | **28** | *L*_i🡪j_ Rukomeshi 🡪 Busia |
|  | **29** | *L*_i🡪j_ IAEA2012 🡪 IAEA2013 |
|  | **30** | *L*_i🡪j_ IAEA2013 🡪 IAEA2012 |
|  | **31** | *L*_i🡪j_ IAEA2012 🡪 Rukomeshi |
|  | **32** | *L*_i🡪j_ Rukomeshi 🡪 IAEA2012 |
|  | **33** | *L*_i🡪j_ IAEA2013 🡪 Rukomeshi |
|  | **34** | *L*_i🡪j_ Rukomeshi 🡪 IAEA2013 |
|  | **35** | *Na* for the population pair Busia-IAEA2012 |
|  | **36** | *Na* for the population pair Busia-IAEA2013 |
|  | **37** | *Na* for the population pair Busia- Rukomeshi |
|  | **38** | *Na* for the population pair IAEA2012- IAEA2013 |
|  | **39** | *Na* for the population pair IAEA2012- Rukomeshi |
|  | **40** | *Na* for the population pair IAEA2013- Rukomeshi |
|  | **41** | *H* for the population pair Busia-IAEA2012 |
|  | **42** | *H* for the population pair Busia-IAEA2013 |
|  | **43** | *H* for the population pair Busia- Rukomeshi |
|  | **44** | *H* for the population pair IAEA2012- IAEA2013 |
|  | **45** | *H* for the population pair IAEA2012- Rukomeshi |
|  | **46** | *H* for the population pair IAEA2013- Rukomeshi |
|  | **47** | *VAR* for the population pair Busia-IAEA2012 |
|  | **48** | *VAR* for the population pair Busia-IAEA2013 |
|  | **49** | *VAR* for the population pair Busia- Rukomeshi |
|  | **50** | *VAR* for the population pair IAEA2012- IAEA2013 |
|  | **51** | *VAR* for the population pair IAEA2012- Rukomeshi |
|  | **52** | *VAR* for the population pair IAEA2013- Rukomeshi |
|  | **53** | maximum likelihood estimates for admixture proportions between Busia and Rukomeshi in IAEA2012 |
|  | **54** | maximum likelihood estimates for admixture proportions between Busia and Rukomeshi in IAEA2013 |

*Na*: mean number of alleles. *H*: mean gene diversity. *VAR*: mean allele size variance. MGW: mean M index. *L*_i🡪j_: mean individual assignment log-likelihoods of individuals from population *i* assigned to population *j*.
